# A three-tiered colloidosomal microreactor for continuous flow catalysis

**DOI:** 10.1038/s41467-021-26381-x

**Published:** 2021-10-20

**Authors:** Hua Wu, Xuanlin Du, Xiaohui Meng, Dong Qiu, Yan Qiao

**Affiliations:** 1 Beijing National Laboratory for Molecular Sciences (BNLMS), Laboratory of Polymer Physics and Chemistry, CAS Research/Education Center for Excellence in Molecular Sciences, Institute of Chemistry, Chinese Academy of Sciences, Beijing, 100190 China; 2 University of Chinese Academy of Sciences, Beijing, 100049 China

**Keywords:** Bioinspired materials, Colloids, Self-assembly

## Abstract

Integrative colloidosomes with hierarchical structure and advanced function may serve as biomimetic microreactors to carry out catalytic reactions by compartmentalizing biological species within semipermeable membranes. Despite of recent progress in colloidosome design, integration of biological and inorganic components into tiered structures to tackle the remaining challenges of biocatalysis is highly demanded. Here, we report a rational design of three-tiered colloidosomes via the Pickering emulsion process. The microreactor consists of crosslinked amphiphilic silica-polymer hybrid nanoparticles as the semipermeable shell, an enzyme-incorporated catalytic sub-layer, and a partially-silicified adsorptive lumen. By leveraging confinement and enrichment effect, we demonstrate the acceleration of lipase-catalyzed ester hydrolysis within the microcompartment of organic-inorganic hybrid colloidosomes. The catalytic colloidosomes are further assembled into a closely packed column for enzymatic reactions in a continuous flow format with enhanced reaction rates. The three-tiered colloidosomes provide a reliable platform to integrate functional building blocks into a biomimetic compartmentalized microreactor with spatially controlled organization and high-performance functions.

## Abstract (web summary)

Integration of biological and inorganic components into hierarchical assembled structures to enhance catalytic reactions remains challenging. Here, the authors report a rational design of three-tiered colloidosomes via the Pickering emulsion process and demonstrate the acceleration of lipase catalyzed ester hydrolysis within a continuous flow reactor packed with the colloidosomes.

## Introduction

Confinement of biomolecules and biochemical reactions within membrane-bound microcompartments is a key step towards the construction of biomimetic microreactors that are capable of mimicking the rudimentary functionality of living cells^1,2^. Towards the localization of enzymatic elements, synthetic strategies primarily take advantage of spontaneous or directed amphiphile assembly in solution or at interface to produce microcompartments such as fatty acid vesicles^3^ and liposomes^4,5^, polymersomes^6–8^, layer-by-layer capsules^9^, and proteinosomes^10,11^. Such bottom-up approaches offer plausible scenarios for the origin of life and have been utilized to demonstrate a diverse range of cell-mimicking functions including enzyme-mediated catalysis^12,13^, deoxyribonucleic acid (DNA) amplification^14^, and gene expression^15^. Recently, cell-sized colloidosomal microcompartments have been developed from self-assembly of colloid particles (e.g., SiO_2_ spherical particles^16^, Janus microgels^17,18^, metal-containing hybrid particles^19,20^, metal–organic frameworks^21^, or magnetic particles^22^) at the oil/water interface via Pickering emulsion^23–26^. These semi-permeable and robust structures comprise ordered aggregates of colloids and have been used to partially mimic the biological membranes^27–30^.

In-housing integrated biological or biomimetic functions with colloidosomal microreactors have been achieved for applications such as gene expression^31^, enzyme reactions^32,33^, artificial cytoskeletal assembly^34^, cell growth and division^35^, and cell communication^36–38^. A central step towards these cell-like entities is the capability of spatially organizing building blocks within confined space and eventually optimizing the performance of the artificial systems^39^. This usually requires well-defined organization of active elements in microcompartments via multiple noncovalent forces^40^. Besides, efficient mass transporting also plays a key role in increasing the efficiency of functional processes within colloidosomes, especially for those involving multiphase reactions^41,42^.

**Fig. 1:**
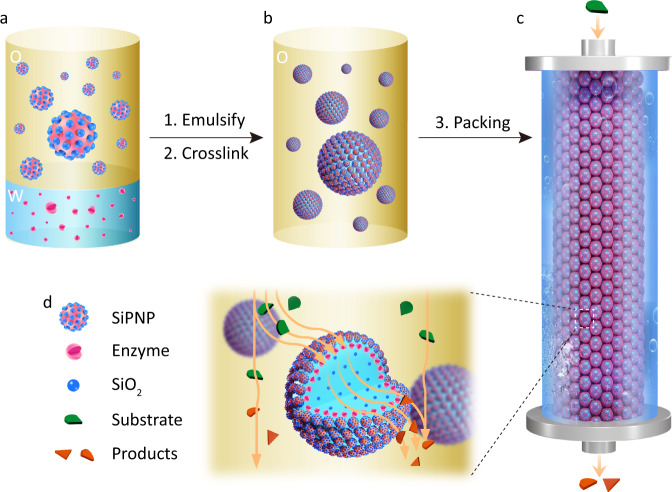
A schematic diagram illustrating the construction of three-tiered colloidosomal microreactors for continuous flow catalysis. **a** A water/oil two-phase co-existing system containing enzymes in the aqueous phase and amphiphilic SiO_2_-polymer patchy nanoparticles (SiPNPs) in isooctanol. **b** Formation of Pickering emulsion stabilized by amphiphilic SiPNPs via mechanical emulsification, which was subsequently crosslinked at the oil/water interface with the addition of tetramethoxysilane (TMOS). The resulting colloidosomes displayed a three-tiered structure containing a hybrid membrane of SiPNPs, an enzyme sub-layer, and a silica-filled interior. **c** A column packed with the three-tiered colloidosomes was prepared for continuous flow catalysis. **d** Schematic of the local flow in a colloidosome-filled column reactor with the mobile influx of enzyme substrates and efflux of catalytic products.

In this work, we aim to integrate distinct building blocks of colloidosomes into cell-like microcompartments with microstructural hierarchy and explore their potential application as microreactors. To this end, we prepared organic–inorganic hybrid capsules comprising closely packed SiO_2_-polymer patchy nanoparticles (SiPNPs) synthesized from a water-in-isooctanol Pickering emulsion (Fig. 1), in which a catalytic enzyme sub-layer was incorporated. The hybrid silica-polymer membrane of colloidosomes was further reinforced by surface crosslinking with tetramethoxysilane (TMOS), which caused the partial silicification of the aqueous interior. All together, we demonstrated the rational design of hierarchical colloidosomes consisting of a hybrid semi-permeable membrane, an enzyme sub-layer, and an aqueous interior filled with adsorptive silica nanoparticles. We further used colloidosomes as biomimetic microreactors and conducted enzyme-catalytic reactions within the membrane-gated microcompartments in bath reaction system and a continuous flow system.

## Results

### Construction of hierarchical three-tiered colloidosomes

**Fig. 2:**
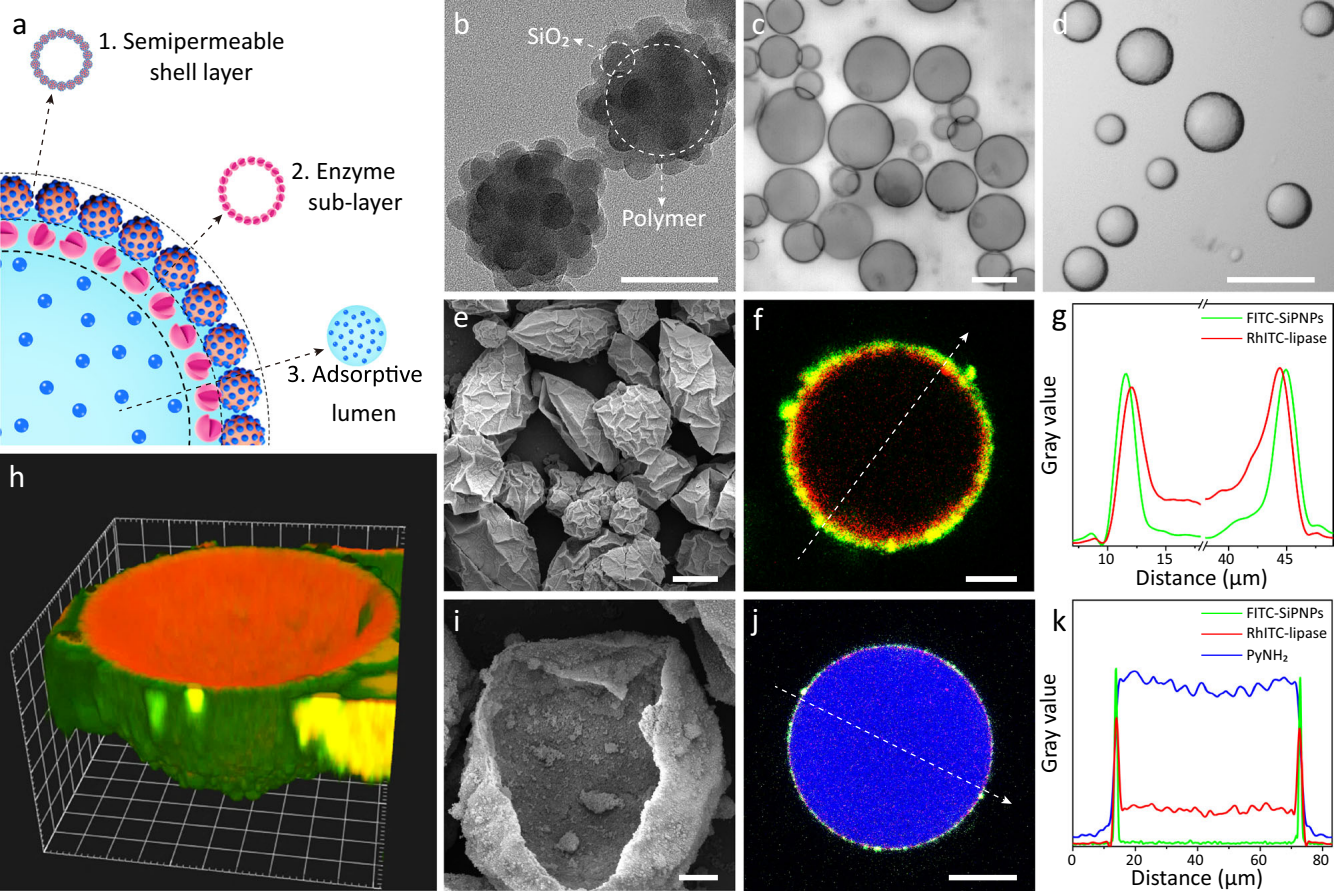
Structural and morphological characterization of the three-tiered colloidosomes. **a** Schematic illustration of a three-tiered colloidosome consisting of a semi-permeable crosslinked SiPNP layer, a catalytic sub-layer of lipase, and an aqueous lumen with adsorptive SiO_2_ nanoparticles. **b** Transmission electron microscopy image of SiPNPs showing the raspberry-like shape of SiO_2_-polymer hybrid nanoparticles. Scale bar: 100 nm. **c**, **d** Optical micrographs of the lipase-loaded non-crosslinked colloidosomes (**c**) and the crosslinked colloidosomes (**d**). Scale bars: 20 μm. **e** SEM image of the crosslinked colloidosomes showing the wrinkled hybrid shells. Scale bar: 20 μm. **f**–**h** CLSM image (**f**), the corresponding fluorescence intensity profiles (**g**), and 3D-reconstructed CLSM image (**h**) were used to identify the SiPNP shell (green fluorescence) and the lipase layer (red fluorescence) of the crosslinked colloidosomes. Scale bar: 20 μm. Grid width in **h**: 4 μm. **i** SEM micrograph of a cracked colloidosome showing a thick hybrid silica-polymer layer and small SiO_2_ nanoparticles in the interior phase. Scale bar: 2 μm. **j**, **k** CLSM image (**j**) and the corresponding fluorescence intensity profiles (**k**) showing the uptake of PyNH_2_ by the crosslinked colloidosomes in isooctanol. Scale bar: 20 μm.

Following the steps shown schematically in Fig. 1, we successfully constructed a colloidosomal microreactor consisting of crosslinked SiPNPs as the semi-permeable shell layer, an inner attached enzyme sub-layer as the catalytic site, and silica nanoparticle-filled adsorptive lumen (Fig. 2a). The patchy SiPNPs were synthesized by one-step emulsion polymerization of 3-(trimethoxysilyl) propyl methacrylate (TPM) in the presence of hydrophilic SiO_2_ nanoparticles (~25 nm)^43^. The discrete hybrid nanoparticles exhibited a raspberry-like morphology and a hydrodynamic diameter of ~125 nm (Fig. 2b and Supplementary Fig. 1a–c). The combination of hydrophilic silica and hydrophobic polymer into SiPNPs gave rise to a colloidal amphiphilicity that offered excellent particle dispersity in both organic solvents (e.g., toluene and isooctanol) and water (Supplementary Fig. 2a). This property further enabled the preparation of toluene-in-water (oil/water) and water-in-isooctanol (water/oil) Pickering emulsions by using SiPNPs as the stabilizing agents (Supplementary Fig. 2b).

To incorporate enzyme into colloidosomes, we incubated an aqueous solution of lipase (1.5 mg mL^−1^ in Tris-HCl buffer) with an isooctanol suspension of amphiphilic SiPNPs (1 mg mL^−1^) to form a water-in-oil Pickering emulsion (~45 μm, Fig. 2c and Supplementary Fig. 3). We observed that the lipase did not lose catalytic activity after complexing with SiPNPs (Supplementary Fig. 4). These enzyme-incorporated microcompartments remained structurally stable for several weeks, whereas emulsion stabilized by lipase alone demulsified within 20 min, indicating the crucial role of the amphiphilic SiPNPs in colloidosome stability (Supplementary Figs. 5–7).

The lipase-loaded colloidosomes were further crosslinked with the addition of TMOS, which generated interconnected silica network between seed nanoparticles at the interface and thereby enhanced the robustness of the membrane (Fig. 2d). Scanning electron microscopy (SEM) image showed a wrinkled continuous silica-polymer shell on air-dried colloidosomes (Fig. 2e), which became smooth and spherical shape at a higher concentration of TMOS (Supplementary Fig. 8). The crosslinked colloidosomes maintained a structural integrality after phase transfer and drying/rehydration cycles without noticeable morphological distortion (Supplementary Figs. 9 and 10).

The spatial distribution of lipase and silica-polymer layers was evident from confocal laser scanning microscopy (CLSM) measurement, in which patchy SiPNP nanoparticles and lipase were specifically labeled with fluorescein isothiocyanate (FITC) and rhodamine isothiocyanate (RhITC), respectively. The FITC/RhITC labeling allowed us to identify the composition of colloidosome membrane by distinguishing the outer FITC-SiPNPs layer (green fluorescence) and inner RhITC-lipase layer (red fluorescence) from the CLSM images (Fig. 2f, g) and the three-dimensional (3D)-reconstructed CLSM images (Fig. 2h and Supplementary Fig. 11)^44^. It was clearly seen from SEM results (Supplementary Fig. 12) that lipase was adsorbed on the inner surface of SiPNP layer, likely due to the hydrophobic effect and charge interaction between lipase and SiPNPs at the oil/water interface^45–47^. The lipase-contained hierarchical structure was demonstrated to be stable, without noticeable leakage of enzyme from colloidosomes (Supplementary Fig. 13). Similar tiered structures were obtained by using amphiphilic enzymes (e.g., urease, glucose oxidase, and horseradish peroxidase). In contrast, trypsin with increased hydrophilicity did not specifically remain at the interface of colloidosomes (Supplementary Fig. 14). In addition, SEM (Fig. 2i) and fluorescence microscopy images (Supplementary Fig. 15) showed the formation of SiO_2_ nanoparticles in the aqueous lumen, which was likely due to the hydrolysis of TMOS inside the colloidosomes. All together, we have demonstrated a general approach to construct three-tiered colloidosomes via the Pickering emulsion process.

We further investigated the performance of colloidosomes for encapsulating a range of biomolecules (Supplementary Fig. 16). Anionic compounds (i.e., calcein and carboxyfluorescein-labeled single-stranded DNA (FAM-ssDNA) and non-ionic molecules (i.e., FITC-dextran) were encapsulated and homogenously distributed within the non-crosslinked colloidosomes, whereas cationic molecules such as rhodamine 6G (Rh6G) and RhITC-poly(diallyldimethylammonium chloride) (RhITC-PDDA) preferred to adsorb at the interface. Upon crosslinking with TMOS, the surface-absorbing compounds were observed to redistribute in the interior of the colloidosomes regardless of the environmental solvents (isooctanol and water).

We also investigated the uptake capability of colloidosomes in isooctanol before and after crosslinking. Hydrophobic compounds such as 1-pyrenemethylamine hydrochloride (PyNH_2_) and fluorescent phospholipid nitro benzoxadiazol-phosphoethanolamine (NBD-PE) cannot be segregated by the non-crosslinked colloidosomes (Supplementary Fig. 17a, b). After crosslinking the shell with TMOS, however, the resultant shell-crosslinked colloidosomes enriched PyNH_2_ and NBD-PE onto the shell (Supplementary Fig. 17c, d). In contrast, the majority of hydrophobic PyNH_2_ and NBD-PE was observed to be sequestered within the aqueous lumen of three-tiered colloidosomes (Fig. 2j, k and Supplementary Fig. 17e, f and Movie 1)^48,49^, indicating that the formation of silica nanoparticles via TMOS hydrolysis was accounted for the molecule uptake of the aqueous lumen.

We further explored the uptake behavior in mixed solutions (e.g., Nile red, NBD-PE, and PyNH_2_ in isooctanol; bovine serum albumin (BSA), dextran, and Hoechst in water) by colloidosomes. It is observed that small hydrophobic (e.g., Nile red, NBD-PE, and PyNH_2_) and hydrophilic (e.g., Hoechst) compounds were efficiently sequestered by the lumen of colloidosome (Supplementary Fig. 18 and Movie 2), macromolecules such as PDDA (200 kDa) and DNA (31 kDa) showed relatively slower penetration through the shell (Supplementary Fig. 19 and Movie 3), whereas amphiphilic molecules (e.g., BSA) and rigid biopolymers (e.g., dextran) were mainly stuck at the shell possibly due to protein–SiPNPs hydrophobic interactions and backbone rigidness-limited diffusion, respectively (Supplementary Figs. 20 and 21, and Movie 4). Taken together, we believe that the hierarchical colloidosome can act as a semi-permeable compartment to selectively sequester biomolecules, which is primarily due to the semi-permeable crosslinked silica-polymer gated membrane and the adsorptive nanoparticle-filled inner cavity.

### Enhanced catalytic reaction within three-tiered colloidosomes

**Fig. 3:**
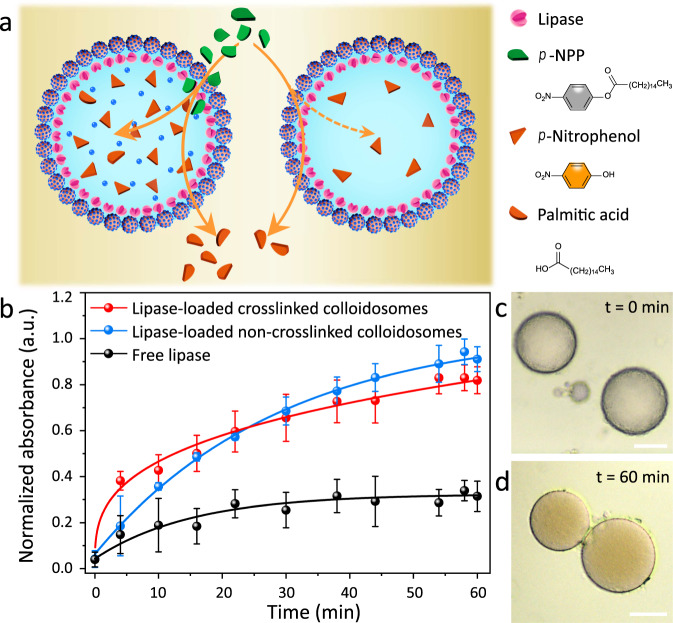
A colloidosome-based microreactor for lipase-catalyzed hydrolysis reactions. **a** Comparison of lipase-catalyzed hydrolysis within the lipase-loaded colloidosomes with and without TMOS crosslinking. **b** Kinetics of *p*-NPP hydrolysis into *p*-nitrophenol catalyzed by lipase-loaded crosslinked colloidosomes (red plot), lipase-loaded non-crosslinked colloidosomes (blue plot), and free lipase (black plot). The loaded lipase for all groups was 0.005 mg. Error bars indicate the SD of three replicating measurements. **c**, **d** Optical microscopy images of the lipase-loaded crosslinked colloidosomes before (**c**) and after (**d**) *p*-NPP hydrolysis, showing the generation of yellow *p*-nitrophenol over time inside the aqueous lumen. Scale bars: 20 μm.

Given that the colloidosomes were able to uptake external molecules efficiently in both oil and water phases, we proposed that the three-tiered colloidosomes may act as microreactors that are able to enrich enzyme substrates for accelerated catalytic reactions. To prove our hypothesis, we examined the hydrolysis of *p*-nitrophenyl palmitate (*p*-NPP) in lipase-loaded colloidosomes before and after crosslinking (Fig. 3a), in which oil-soluble *p*-NPP was hydrolyzed into a yellow product *p*-nitrophenol with a characteristic absorption peak at 410 nm. Figure 3b showed a rapid increase of *p*-nitrophenol absorption intensity at the initial stage (*t* < 24 min) in the lipase-loaded crosslinked colloidosomes, and a relatively slow increase thereafter. Compared to crosslinked colloidosomes, the hydrolysis reaction with lipase-loaded non-crosslinked colloidosomes was slower at the initial stage. The reaction catalyzed by free lipase was found to be even slower, highlighting the importance of colloidosomal microreactor in boosting confined reactions. The underline reason may be the greatly enlarged reaction interfacial area of colloidosomal microreactor^50^. The occurrence of lipase-catalyzed *p*-NPP hydrolysis within the confined colloidosomal microreactor was also confirmed by optical microscopy, where colorless microcompartments turned into yellow after reaction (Fig. 3c, d). The high catalytic reaction rate in crosslinked colloidosomes was likely due to the enrichment of the substrate *p*-NPP by silica nanoparticles in the adsorptive lumen. The importance of adsorptive silica was highlighted in Supplementary Fig. 22, where the initial reaction rate was found to increase with the amount of TMOS (precursor of silica). After the initial stage, the reaction slowed down due to the accumulation of products (nitrophenol and palmitic acid) within colloidosomes.

### Ester hydrolysis in a column reactor packed with three-tiered colloidosomes

**Fig. 4:**
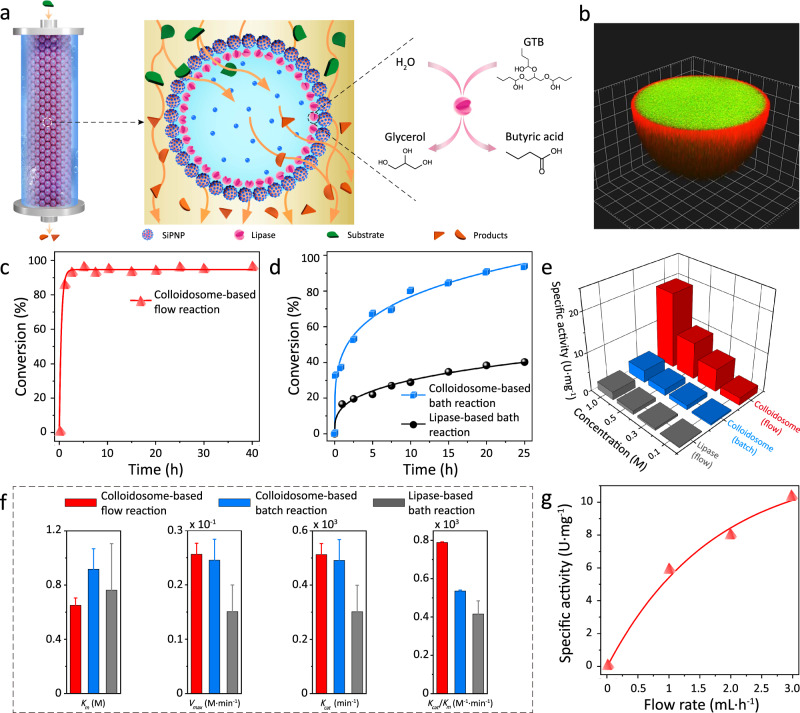
Lipase-catalyzed GTB hydrolysis in a column reactor packed with three-tiered colloidosomes. **a** Scheme showing the continuous influx of lipase substrates (i.e., GTB) into the three-tiered colloidosomal microreactors in a column containing mobile oil phase, where the ester hydrolysis took place on the catalytic layer to generate the product efflux (i.e., glycerol and butyric acid). **b** 3D-reconstructed image of the three-tiered colloidosomes with RhITC-lipase catalytic layer (red fluorescence) and the substrate-enriched lumen (NBD-PE here as example, green fluorescence). Grid width: 4 μm. **c**, **d** Time-dependent conversion for GTB (0.5 M in isooctanol) hydrolysis in the colloidosome-based flow reaction, loaded lipase: 0.84 mg (**c**); colloidosome- and lipase-based bath reactions, loaded lipase: 0.21 mg (**d**). A comparison revealed the high efficiency and stability of the flow system. The concentration of GTB was calibrated according to the constructed calibration plots and corresponding equations (Supplementary Fig. 23). **e** Specific activity (SA) values of lipase in the colloidosome-based flow reaction at steady state (1 mL h^−1^), and colloidosome- and lipase-based bath reactions within 20 h, respectively. **f** Comparison of the Michaelis–Menten kinetic parameters, *k*_m_, *v*_max_ and *k*_cat_, *k*_cat_/*k*_m_, for the colloidosome-based flow reaction, and colloidosome- and lipase-based bath reactions, respectively. Error bars indicate the SD of three replicating measurements. **g** SA plot of lipase in the colloidosome-based flow reaction against flow rate (substrate concentration of 0.3 M), showing the larger SA resulted from the higher flow rate of the oil mobile phase. The column temperature for all experiments in the flow system was set as 40 °C. The flow rate for all flow reactions was 1 mL h^−1^, except for **g**.

In order to avoid slow reaction after the initial stage, we proposed a continuous flow system that allow for removing the hydrolysis products and thereby maintaining long-term catalytic activity of colloidosomes. As shown in Fig. 4a, the column was packed with the three-tiered colloidosomal microreactors (~45 μm), which can offer sufficient void channels for the oil flow to pass through and reduce the pressure drop in the column. With the continuous influx of hydrophobic glyceryl tributyrate (GTB) through the lipase-containing colloidosomal microreactors, the reaction products (glycerol and butyric acid) can be efficiently removed by the efflux, resulting in a rapid hydrolysis rate under the non-equilibrium reaction condition. The 3D-reconstructed CLSM image in Fig. 4b revealed the hierarchical structure of colloidosomes, in which the red fluorescence represented the catalytic layer of RhITC-lipase and the green fluorescence was the adsorptive silica-dispersing lumen.

We further evaluated the reaction efficiency in the colloidosome-based flow reaction, and colloidosome- and lipase-based bath reactions. As shown in Fig. 4c, d, the reaction rate of GTB hydrolysis in colloidosome-embedded flow systems was observed to be higher than those in the bath systems of lipase-loaded colloidosomes and lipase. GTB conversion in the continuous flow reactor packed with lipase-loaded three-tiered colloidosomes reached a steady conversion of ~95% with the specific activity (SA) of 9.54 U mg^−1^, higher than that of colloidosome-based reaction (~52%, 1.67 U mg^−1^) and lipase-based bath reaction (~19%, 0.92 U mg^−1^), collaborated by the comparison of initial SA for flow and batch systems (Supplementary Fig. 24). We proposed that the coupling of colloidosomes with a continuous flow system greatly eliminated the product inhibition of the enzymatic reaction, which led to a long-term high-efficient catalysis mode.

We further analyzed the lipase SA and reaction kinetic parameters in the three-tiered colloidosome-based flow reactions. As shown in Fig. 4e, the SA values of lipase in the flow system surged upon increasing the GTB concentration, while this effect was less significant in the bath reaction system. This is possibly due to the fast product removal in colloidosome-based flow system. These results indicated that the flow reaction system possessed better tolerance at high substrate concentrations (Supplementary Fig. 25). In addition, we determined the kinetic parameters of lipase-mediated reactions in the flow and bath systems by changing the concentrations of substrates (Supplementary Fig. 26). Figure 4f presented the apparent Michaelis–Menten constant *k*_m_, the maximal reaction rate *v*_max_ (rate of the reaction when the active sites of the enzyme are saturated with substrate), the turnover number *k*_cat_, and the ratio of *k*_cat_ and *k*_m_ (*k*_cat_/*k*_m_, representing the general enzymatic efficiency)^51^. The *k*_m_ value for the flow system was determined to be 0.65 ± 0.05 M, close to that for batch systems, suggesting a similar affinity between the enzyme and reactant due to the homologous reaction environment for these reaction systems^50^. *V*_max_ of the flow system was estimated to be 0.26 M min^−1^, nearly double as high as that of the bath systems. Moreover, *k*_cat_ and *k*_cat_/*k*_m_ of the flow system were 1.85 times and twice as high as those corresponding to the bath reactions, respectively, which all together demonstrated the increased reaction efficiency of the colloidosome-based flow reaction. The reaction enhancement could be attributed to both the three-tiered microarchitecture of colloidosomes capable of enriching substrates within confined space and the continuous flow system that enabled the removal of reaction products. On the basis of this hypothesis, the SA values in the flow system can be further improved by increasing the flow rate of the mobile phase (Fig. 4g).

## Discussion

In conclusion, we constructed three-tiered catalytic colloidosomes comprising of a semi-permeable membrane with hybrid silica-polymer layer, an enzyme sub-layer, and an internal aqueous lumen capable of enriching biomolecules. The microcompartments showed selective sequestration of molecules ranging from organic dyes, proteins, polysaccharide, and DNA, which empower the acceleration of enzymatic reactions. By packing the three-tiered colloidosomes into a flow column, we were able to alleviate the inhibition effect arising from the accumulation of products by using a continuous flow reaction format. Our results demonstrate a sophisticated approach to integrate multiple building blocks into a hierarchical compartment with optimized functionalities, which could potentially find applications in broad fields such as microreactors, biomimetics, synthetic biology, and nanomedicine. The combination of hierarchical structures along with the non-equilibrium reaction represents a viable approach towards cell-like microcompartments and catalytic microreactors.

## Methods

### Synthesis of amphiphilic raspberry-like SiO_2_-TPM composite nanoparticles (SiPNPs)

Typically, 0.56 mL of ludox silica TM40 suspension was shaken and added into 40 mL Milli-Q water in a 100 mL three-necked flask, followed by adding 0.5 mL of TPM. The mixture was stirred for 24 h to form an emulsion, then deoxygenated by bubbling nitrogen for 30 min and heated to 70 °C. Polymerization was initiated by adding 10 mg of potassium peroxydisulfate and the mixture was incubated at 70 °C for another 24 h. The resultant suspension was centrifuged at 4800 × *g* for 5 min and the supernatant was discarded. The products were washed with ethanol for three times and dried in vacuum for further use.

### Preparation of the three-tiered colloidosomal microreactors

Typically, 1.0 mg of the as-prepared SiPNPs was added into 1.0 mL of isooctanol in a 2 mL centrifuge tube. The mixture was dispersed by homogenization with an UltraTurrax T10 homogenizer for 5 min at 6000 r.p.m. To this mixture, 70 μL of lipase aqueous solution (1.5 mg mL^−1^ in Tris-HCl buffer, the concentration of lipase to load the colloidosomes) was added and the suspension was homogenized for 1 min at 3000 r.p.m. to yield a Pickering emulsion. The water/oil volume ratio of the system was 0.07 and the concentration of SiPNPs in oil was set to 1 mg mL^−1^, respectively. Afterwards, 20 μL of TMOS was added to 1.07 mL of the preformed Pickering emulsion to crosslink the SiPNPs at oil–water interface. The mixture was gently shaken for 3 min and left unstirred overnight at room temperature.

### Molecule encapsulation within colloidosomal microreactors

Colloidosomes with encapsulated molecules were prepared following the colloidosome preparation procedures, except that both lipase and fluorescent molecules were included in the aqueous solution. Typically, 10 μL of Rh6G (0.2 mM), RhITC-PDDA (0.5 mg mL^−1^), calcein (0.2 mM), FAM-ssDNA (100 μM, Supplementary Table 1), FITC-dextran (0.5 mg mL^−1^), or RhITC-BSA (0.5 mg mL^−1^) was added to 60 μL of lipase solution. This solution was mixed with isooctanol containing amphiphilic raspberry-like SiPNPs (1 mg mL^−1^, 1 mL) and homogenized with 3000 r.p.m. for 1 min.

### Molecular uptake of the three-tiered colloidosomal microreactors

For uptake tests in oil phase, the solution of PyNH_2_ (1 mg mL^−1^, in dimethyl sulfoxide) or NBD-PE (1 mM, in methanol) was added into the dispersion of colloidosomes in isooctanol at a volume ratio of 0.02, respectively. The uptake experiments were monitored by CLSM after 5 min of mixing.

For uptake tests in water phase, the aqueous solution of Rh6G (0.2 mM), Hoechst (20 μM), RhITC-PDDA (0.5 mg mL^−1^), calcein (0.02 mM), FAM-ssDNA (100 μM), FITC-dextran (0.5 mg mL^−1^), or RhITC-BSA (0.05 mg mL^−1^) was added into the aqueous colloidosome dispersions at a volume ratio of 0.02, respectively. The uptake experiments were monitored by CLSM.

For uptake tests of mixture in oil and water phase, all dye solutions were premixed for further use. Typically, 10 μL of Nile red (0.5 mg mL^−1^, in methanol), NBD-PE (1 mM, in methanol), and PyNH_2_ (1 mg mL^−1^, in dimethyl sulfoxide) were premixed and 1 μL of the mixture was added into 50 μL of colloidosome oil dispersion. Likewise, 10 μL of RhITC-BSA (0.05 mg mL^−1^), FITC-dextran (0.5 mg mL^−1^), and Hoechst (20 μM) were premixed and 1 μL of the mixture was added into 50 μL of colloidosome aqueous dispersion.

### Lipase-catalyzed *p*-NPP hydrolysis within colloidosomes in bath reaction system

For colloidosomes, 50 μL of as-prepared colloidosomes in isooctanol with encapsulated lipase (1.5 mg mL^−1^ in 50 mM Tris-HCl buffer pH 8.0) was mixed with 50 μL *p*-NPP (15 mg mL^−1^ in isooctanol) in a 96-well microplate (Costar) with a transparent flat bottom. For free enzyme, 3.21 μL of lipase solution (1.5 mg mL^−1^) was mixed with 46.79 μL isooctanol and 50 μL p-NPP (15 mg mL^−1^). The absorbance of reaction mixture at 410 nm was recorded for every time (300 s) on a plate reader (CLARIO star plus, BMG Labtech), which is equipped with a temperature controller to maintain the reaction system at 40 °C.

### Lipase-catalyzed GTB hydrolysis by colloidosomes in bath reaction system

Typically, 2 mg of SiPNPs was added into 2 mL of isooctanol and homogenized for 5 min at 6000 r.p.m. Next, 140 μL of lipase solution (1.5 mg mL^−1^ in 50 mM Tris-HCl buffer pH 8.0) was then added followed by homogenization (3000 r.p.m., 1 min) and crosslinking (40 μL, >6 h) processes. The colloidosomes settled down from the dispersion and were consequently transferred to 1 mL of GTB in isooctanol (e.g., 0.5 M) to conduct the enzyme-catalytic reaction with rotating mixer (Kylin-Bell Lab Instruments, BE-1100, China). The temperature of the reaction system was maintained at 40 °C using a constant temperature incubator (Bluepard LRH-150, China). Aliquots of the solution were taken at intervals for monitoring conversions by gas chromatography-mass spectrometry (GC-MS, SHIMADZU QP2010, Japan) analysis (Supplementary Figs. 27–29).

### Lipase-catalyzed GTB hydrolysis by colloidosomes in continuous flow reaction system

Typically, a suspension of the lipase-encapsulating colloidosomes (e.g., 10 mL) in isooctanol was added into a column reactor (0.8 cm of inner diameter) containing a filter disc (~15 μm in pore diameter). A solution of GTB (e.g., 0.5 M) in isooctanol was pumped as mobile phase through the inner tube of the column reactor at a given flow rate (e.g., 1 mL h^−1^). The column temperature was kept at 40 °C by the peripheral casing containing water with constant temperature. The outflow was collected at intervals (e.g., 5 h at steady state) for GC-MS analysis.

The SA of lipase was determined as the amount of substrate converted per min by enzyme (i.e., μmol min^−1^ mg^−1^). SA values were calculated at the steady state in continuous flow reaction, within the first 30 min and over 20 h in bath reactions for further analysis.

## Supplementary information

Supplementary Information

Description of Additional Supplementary Files

Supplementary Movie 1

Supplementary Movie 2

Supplementary Movie 3

Supplementary Movie 4

## Data Availability

All data needed to evaluate the conclusions in the paper are present in the paper and/or the Supplementary Materials.

## References

[1] Szostak JW, Bartel DP, Luisi PL (2001). Synthesizing life. Nature.

[2] Mann S (2012). Systems of creation: the emergence of life from non-living matter. Acc. Chem. Res..

[3] Bianco CD, Torino D, Mansy SS (2014). Vesicle stability and dynamics: an undergraduate biochemistry laboratory. J. Chem. Educ..

[4] Stano P, Carrara P, Kuruma Y, Souza T, Luisi PL (2011). Compartmentalized reactions as a case of soft-matter biotechnology: synthesis of proteins and nucleic acids inside lipid vesicles. J. Mater. Chem..

[5] Nourian Z, Danelon C (2013). Linking genotype and phenotype in protein synthesizing liposomes with external supply of resources. ACS Synth. Biol..

[6] Feng AC, Yan Q, Zhang HJ, Peng L, Yuan JY (2014). Electrochemical redox responsive polymeric micelles formed from amphiphilic supramolecular brushes. Chem. Comm..

[7] Huo M (2017). Tailoring the multicompartment nanostructures of fluoro-containing ABC triblock terpolymer assemblies via polymerization-induced self-assembly. Macromolecules.

[8] Ji, Y., Mu, W., Wu, H. & Qiao, Y. Directing transition of synthetic protocell models via physicochemical cues-triggered interfacial dynamic covalent chemistry. *Adv. Sci*. **8**, 2101187 (2021). 10.1002/advs.202101187 PMC8456217 34319646

[9] Duan L (2007). Hemoglobin protein hollow shells fabricated through covalent layer-by-layer technique. Biochem. Biophys. Res. Commun..

[10] Huang X (2013). Interfacial assembly of protein-polymer nano-conjugates into stimulus-responsive biomimetic protocells. Nat. Commun..

[11] Mu WJ (2021). Membrane-confined liquid-liquid phase separation toward artificial organelles. Sci. Adv..

[12] Martin N (2018). Antagonistic chemical coupling in self-reconfigurable host-guest protocells. Nat. Commun..

[13] Qiao Y, Li M, Qiu D, Mann S (2019). Response-retaliation behavior in synthetic protocell communities. Angew. Chem. Int. Ed..

[14] Kurihara K (2015). A recursive vesicle-based model protocell with a primitive model cell cycle. Nat. Commun..

[15] Mansy SS (2008). Template-directed synthesis of a genetic polymer in a model protocell. Nature.

[16] Wang HL, Zhu XM, Tsarkova L, Pich A, Moller M (2011). All-silica colloidosomes with a particle-bilayer shell. ACS Nano.

[17] Suzuki D, Tsuji S, Kawaguchi H (2007). Janus microgels prepared by surfactant-free Pickering emulsion-based modification and their self-assembly. J. Am. Chem. Soc..

[18] Tsuji S, Kawaguchi H (2008). Thermosensitive Pickering emulsion stabilized by poly(N-isopropylacrylamide)-carrying particles. Langmuir.

[19] He J, Liu YJ, Babu T, Wei ZJ, Nie ZH (2012). Self-assembly of inorganic nanoparticle vesicles and tubules driven by tethered linear block copolymers. J. Am. Chem. Soc..

[20] Faria J, Ruiz MP, Resasco DE (2010). Phase-selective catalysis in emulsions stabilized by Janus silica-nanoparticles. Adv. Synth. Catal..

[21] Sun YY (2019). Hierarchically porous and water-tolerant metal-organic frameworks for enzyme encapsulation. Ind. Eng. Chem. Res..

[22] Rodriguez-Arco L, Li M, Mann S (2017). Phagocytosis-inspired behaviour in synthetic protocell communities of compartmentalized colloidal objects. Nat. Mater..

[23] Dinsmore AD (2002). Colloidosomes: selectively permeable capsules composed of colloidal particles. Science.

[24] Lin Y, Skaff H, Emrick T, Dinsmore AD, Russell TP (2003). Nanoparticle assembly and transport at liquid-liquid interfaces. Science.

[25] Nie ZH, Petukhova A, Kumacheva E (2010). Properties and emerging applications of self-assembled structures made from inorganic nanoparticles. Nat. Nanotechnol..

[26] Cui MM, Emrick T, Russell TP (2013). Stabilizing liquid drops in nonequilibrium shapes by the interfacial jamming of nanoparticles. Science.

[27] Li M, Harbron RL, Weaver JVM, Binks BP, Mann S (2013). Electrostatically gated membrane permeability in inorganic protocells. Nat. Chem..

[28] Cai ZY (2019). Mussel-inspired pH-switched assembly of capsules with an ultrathin and robust nanoshell. ACS Appl. Mater. Inter.

[29] Crossley S, Faria J, Shen M, Resasco DE (2010). Solid nanoparticles that catalyze biofuel upgrade reactions at the water/oil interface. Science.

[30] Bollhorst T, Rezwan K, Maas M (2017). Colloidal capsules: nano- and microcapsules with colloidal particle shells. Chem. Soc. Rev..

[31] Li M, Green DC, Anderson JLR, Binks BP, Mann S (2011). In vitro gene expression and enzyme catalysis in bio-inorganic protocells. Chem. Sci..

[32] Wei LJ, Zhang M, Zhang XM, Xin HC, Yang HQ (2016). Pickering emulsion as an efficient platform for enzymatic reactions without stirring. ACS Sustain. Chem. Eng..

[33] Wu CZ, Bai S, Ansorge-Schumacher MB, Wang DY (2011). Nanoparticle cages for enzyme catalysis in organic media. Adv. Mater..

[34] Zhu SP (2020). Rapid multi-level compartmentalization of stable all-aqueous blastosomes by interfacial aqueous phase separation. ACS Nano.

[35] Li M, Huang X, Mann S (2014). Spontaneous growth and division in self-reproducing inorganic colloidosomes. Small.

[36] Qiao Y, Li M, Booth R, Mann S (2017). Predatory behaviour in synthetic protocell communities. Nat. Chem..

[37] Lentini R (2014). Integrating artificial with natural cells to translate chemical messages that direct *E. coli* behaviour. Nat. Commun..

[38] Lentini R (2017). Two-way chemical communication between artificial and natural cells. ACS Cent. Sci..

[39] Williams DS, Patil AJ, Mann S (2014). Spontaneous structuration in coacervate-based protocells by polyoxometalate-mediated membrane assembly. Small.

[40] Gobbo P (2020). Catalytic processing in ruthenium-based polyoxometalate coacervate protocells. Nat. Commun..

[41] Zhang XM (2019). Pickering emulsion-derived liquid-solid hybrid catalyst for bridging homogeneous and heterogeneous catalysis. J. Am. Chem. Soc..

[42] Zhang M (2016). Compartmentalized droplets for continuous flow liquid liquid interface catalysis. J. Am. Chem. Soc..

[43] Wu T (2018). Amphiphilic bioactive filler for acrylic bone cement to enhance its cell adhesion. J. Biomed. Nanotechnol..

[44] Wu H, Qiao Y (2021). Microscopy techniques for protocell characterization. Polym. Test..

[45] Malmsten M (1995). Ellipsometry studies of the effects of surface hydrophobicity on protein adsorption. Colloids Surf. B.

[46] Lord MS (2006). The effect of silica nanoparticulate coatings on serum protein adsorption and cellular response. Biomaterials.

[47] Lundqvist M, Sethson I, Jonsson BH (2004). Protein adsorption onto silica nanoparticles: conformational changes depend on the particles’ curvature and the protein stability. Langmuir.

[48] Wu Z (2004). Organic dye adsorption on mesoporous hybrid gels. Chem. Eng. J..

[49] Parida SK, Dash S, Patel S, Mishra BK (2006). Adsorption of organic molecules on silica surface. Adv. Colloid Interface Sci..

[50] Zhang M (2017). Ionic liquid droplet microreactor for catalysis reactions not at equilibrium. J. Am. Chem. Soc..

[51] Nadar SS, Rathod VK (2018). Encapsulation of lipase within metal-organic framework (MOF) with enhanced activity intensified under ultrasound. Enzym. Microb. Technol..

