# Supplementary material for: A three-tiered colloidosomal microreactor for continuous flow catalysis
Source: Nat Commun. 2021 Oct 20;12:6113. doi: 10.1038/s41467-021-26381-x (PMC8528827; doi:10.1038/s41467-021-26381-x)
Supplement: Supplementary file 1 — Supplementary Information [file 41467_2021_26381_MOESM1_ESM.pdf]

Supplementary Information for

A three-tiered colloidosomal microreactor for continuous flow catalysis

Hua Wu[1,2], Xuanlin Du[1], Xiaohui Meng[1,2], Dong Qiu[1,2]*, Yan Qiao[1,2]*

[1]Beijing National Laboratory for Molecular Sciences (BNLMS), Laboratory of Polymer Physics and Chemistry, CAS Research/Education Center for Excellence in Molecular Sciences, Institute of Chemistry, Chinese Academy of Sciences, Beijing 100190, China

[2]University of Chinese Academy of Sciences, Beijing 100049, China

* Email: dqiu@iccas.ac.cn, yanqiao@iccas.ac.cn

Supplementary methods

Materials. The following chemicals were used as received: ludox silica TM40 (25 nm, Sigma-Aldrich), 3-(trimethoxysilyl)propyl methacrylate (TPM, Alfa Aesar, 97%), isopentyl acetate (PEA, Sinopharm Chemical Reagent, AR), potassium peroxydisulfate (KPS, Alfa Aesar, 97%), tetramethoxysilane (TMOS, Aladdin, 98%), (3-aminopropyl)triethoxysilane (APTES, Aladdin, 99%), ammonia (25 wt% in water, Beijing Chemical Works, AR), 2-ethyl-1-hexanol (isooctanol, Aldrich, ≥ 99.5%), lipase (from *Aspergillus oryzae*, Aladdin), urease (from *Canavalia ensiformis*, Sigma-Aldrich), horseradish peroxidase (HRP, from horseradish, Sigma-Aldrich), glucose oxidase (GOx, from *Aspergillus niger*, Sigma-Aldrich), trypsin (from bovine pancreas, Sigma-Aldrich), Trizma® hydrochloride (Tris-HCl, crystalline powder, Sigma-Aldrich, ≥ 99%), *p*-nitrophenyl palmitate (*p*-NPP, powder, Sigma-Aldrich, ≥ 98% TLC), glyceryl tributyrate (GTB, Aladdin, 98%), bovine serum albumin (BSA, M_w = 66,400 Da, Sigma-Aldrich, ≥ 98%), fluorescein isothiocyanate (FITC, Sigma-Aldrich, ≥ 90% HPLC), rhodamine isothiocyanate (RhITC, mixed isomer, Sigma-Aldrich), calcein (Sigma-Aldrich), rhoamine 6G (Rh6G, Sigma-Aldrich), 1-pyrenemethylamine hydrochloride ($PyNH_2$, Sigma-Aldrich, 95%), Hoechst 33258 pentahydrate (bis-benzimide) (Hoechst, Invitrogen), nitro benzoxadiazol-phosphoethanolamine (NBD-PE, powder,

Avanti), fluorescein isothiocyanate-dextran (FITC-dextran, M_w = 70,000, FITC : glucose = 1 : 250, Sigma-Aldrich).

Single-stranded DNA oligonucleotides (99 nucleotides in length) with 56-FAM (carboxy fluorescein) modifications (FAM-ssDNA) were purchased from Integrated DNA Technologies Inc., Belgium.

Phase transfer of the three-tiered colloidosomes. The crosslinked colloidosomes were transferred from the oil phase into aqueous phase using the following procedures. Firstly, the isooctanol phase at the top of the colloidosome dispersion was removed and ethanol was added. The suspension was then centrifuged at 800 xg for 1 min, and the supernatant was discarded. After that, the colloidosomes were washed successively with aqueous solution of ethanol at the volume fractions of 100%, 70%, 50%, 30% and water, respectively, to remove isooctanol thoroughly.

Fluorescent labeling of SiPNPs. Typically, 3.0 mg of as-prepared SiPNPs were dispersed in 2.0 mL ethanol containing 10 μL ammonia. After sonicating for 1 min, 10 μL of APTES ethanolic solution (5.0 $mg\cdot mL^{-1}$) was added and the mixture was stirred (250 rpm) for 24 h at 50 ℃. Subsequently, 10 μL FITC in dimethyl sulfoxide (DMSO, 1.0 $mg\cdot mL^{-1}$) was added and the dispersion was stirred for another 6 h. The resultant mixture was centrifugated at 4800 xg for 5 min and washed with ethanol for three times. FITC-labelled SiPNPs in ethanol were dried for further use.

Fluorescent labeling of lipase and BSA. Typically, 10.0 mg lipase or BSA was added to 5.0 mL of freshly prepared sodium carbonate buffer solution (pH = 9). Consequently, 100 μL of RhITC dissolved in DMSO (1.0 $mg\cdot mL^{-1}$) was added for every 5 μL under gentle stirring. The mixture was stored at 4 ℃ in dark for 8 h and purified by dialysis (Medicell dialysis tubing, MWCO 12,000-14,000 Da) against Milli-Q water to remove the excess RhITC. The solution was freeze dried to obtain the product.

Optical and fluorescence microscopy. Optical microscopy experiments were performed on an inverted microscope (Leica DMi8, Germany) with a 10× lens and a 100× oil immersion lens and a metallurgical

microscope (Leica DM4M, Germany) with 10× and 20× lens. Fluorescence microscopy experiments were performed on a confocal laser scanning microscope (CLSM, Zeiss LSM880, Germany) with an immersion oil 60× lens. Fluorophores were excited using specific filters with the following excitation (λ_{ex}) and emission wavelength (λ_{em}): $PyNH_2$, λ_{ex} = 405 nm and λ_{em} = 410 - 484 nm; Hoechst, λ_{ex} = 405 nm and λ_{em} = 426 - 680 nm; FITC, λ_{ex} = 488 nm and λ_{em} = 495 - 540 nm; Rh6G, λ_{ex} = 514 nm and λ_{em} = 521 - 699 nm; RhITC, λ_{ex} = 543 nm and λ_{em} = 564 - 665 nm. Image analysis was performed with Image J software.

Scanning electron microscopy (SEM). SEM images were acquired using a S - 4800 microscope (Hitachi, Japan) at 15 kV. The SiPNP colloidal suspension was dropped onto a Silicon wafer and excess solution was removed with a piece of filter paper. The samples were dried at 60 °C overnight and coated with gold through ion sputtering before SEM observation. For the characterization of colloidosome fragments, all procedures were the same except for the pre-treatment of the colloidosome dispersion with sonication for 2 min.

Transmission electron microscopy (TEM). TEM experiments were conducted on JEM 2200FS and JEM 1011 microscopes at 200 kV (JEOL, Japan). Typically, a droplet of sample solution (5 μL) was applied to a carbon film-covered 400 mesh grid (TIANLD T10044, film thickness of 25-30nm, China) fixed on a tweezer. Excess solution was removed with a piece of filter paper. The samples were dried under ambient conditions.

Zeta potential and dynamic light scattering (DLS). Zeta potential and DLS measurements were carried out on Zetasizer Nano-ZS instrument (Malvern, UK) equipped with an internal Peltier temperature controller. Typically, 1 mL of sample was sonicated for 5 min and injected into a DTS1070 folded capillary cell or quartz cuvette for zeta potential measurement or DLS measurement, respectively. Experiments were then carried out at 25 ℃. Average values were obtained from three measurements.

Ultraviolet-visible absorption tests (UV-vis). UV-Vis absorption for *p*-nitrophenol (product of *p*-NPP hydrolysis) at 410 nm was determined by using a plate reader (CLARIO star plus, BMG Labtech).

Gas chromatography-mass spectrometer (GC-MS) analysis. GC-MS analysis was carried out on a QP2010 analyzer (SHIMADZU, Japan) with DB-5MS as column. The following conditions were used for GC-MS test: injector temperature of 250 ℃, heating rate of 20 $℃\cdot min^{-1}$ and split ratio of 10:1.

Supplementary Figures

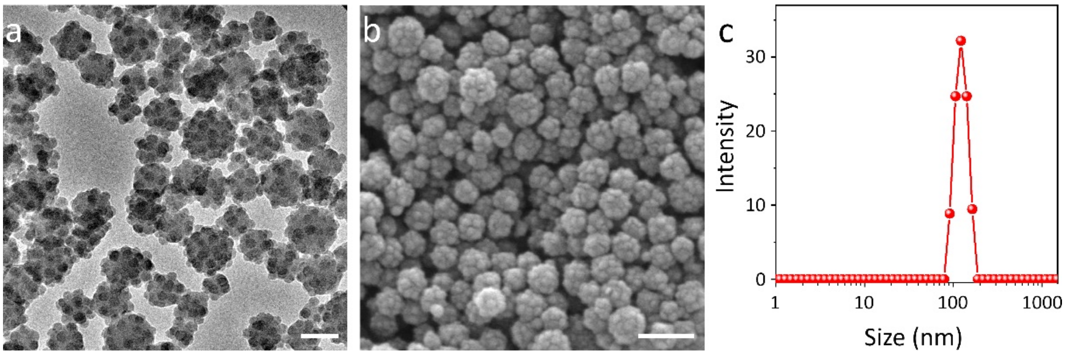

Supplementary Fig. 1. The characterization of the as-synthesized SiPNPs. (**a**) TEM and (**b**) SEM images of SiPNPs. Scale bars: 100 nm. (**c**) DLS showing the size distribution of SiPNPs (1 mg·mL^{-1}) in water at room temperature and the mean size of SiPNPs was determined to be 125 ± 5 nm.

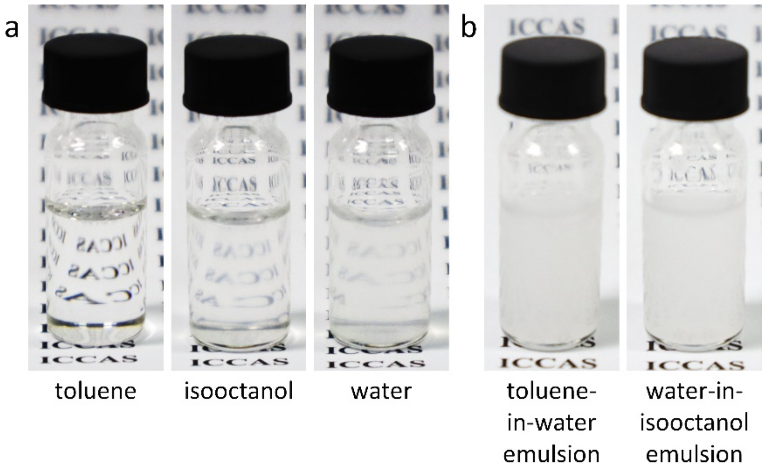

Supplementary Fig. 2. Amphiphilic property of SiPNPs. (**a**) Photographs of SiPNP suspensions (1 mg·mL^{-1}) in toluene (left), isooctanol (middle) and water (right), showing the good dispersity of SiPNPs in these three solvents. (**b**) Photographs of the toluene-in-water (left, oil/water volume ratio was 0.07) and water-in-isooctanol (right, water/oil volume ratio was 0.07) Pickering emulsions stabilized by the as-prepared SiPNPs (1 mg·mL^{-1}).

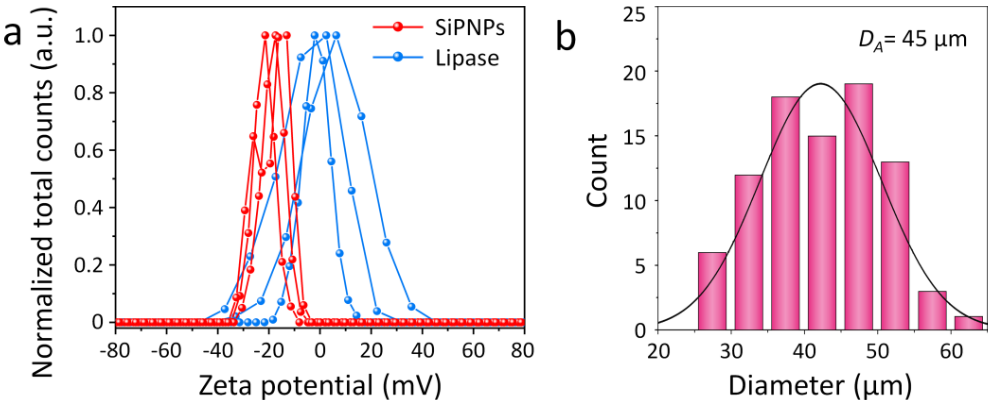

Supplementary Fig. 3. Zeta-potential of lipase, and SiPNPs, and size distribution of colloidosomes. (**a**) Zeta potential of SiPNPs (1 mg·mL^{-1}) and lipase (0.15 mg·mL^{-1}, in tris-HCl buffer, pH = 8.0) solutions at room temperature were determined to be negative charged (-22 ± 0.5 mV) and close to neutral, respectively. (**b**) Diameter distribution of colloidosomes stabilized by SiPNPs was determined to be 45 μm from Gaussian fitting.

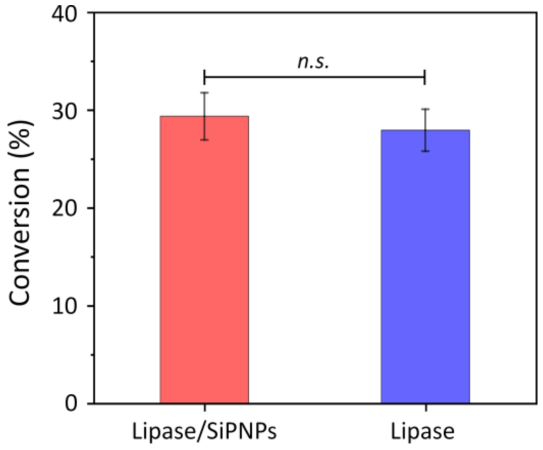

Supplementary Fig. 4. GTB (0.5 M in isooctanol) hydrolysis within 30 min in two-phase system containing lipase/SiPNPs or lipase, respectively. The error bars represented standard deviation of three replicating measurements.

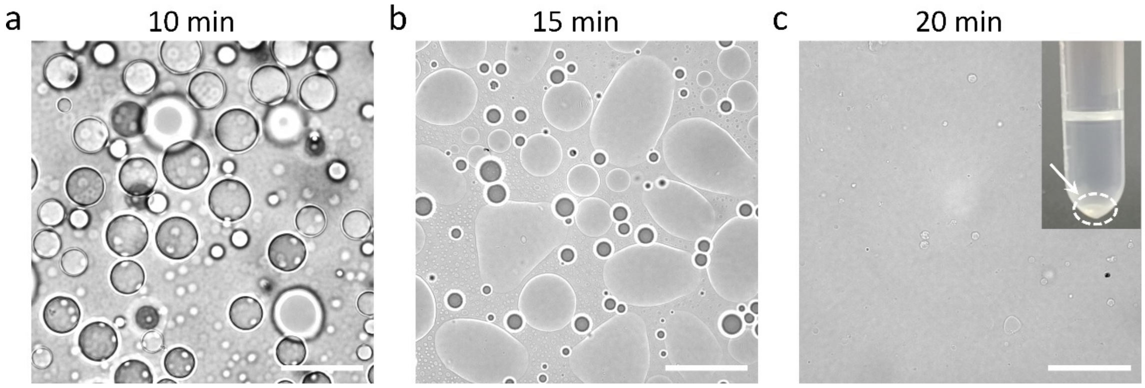

Supplementary Fig. 5. Optical micrographs of water-in-isooctanol emulsion stabilized by lipase alone at (**a**) 10 min, (**b**) 15 min and (**c**) 20 min after homogenization. The lipase concentration was 1.5 mg·mL^{-1}, and the water/oil volume ratio was 0.07. The as-prepared emulsion demulsified within 20 min due to the weak emulsifying capacity of lipase. The inset showed a demulsified two-phase system with water phase settled at the bottom after 30 min. Scale bars: 20 μm.

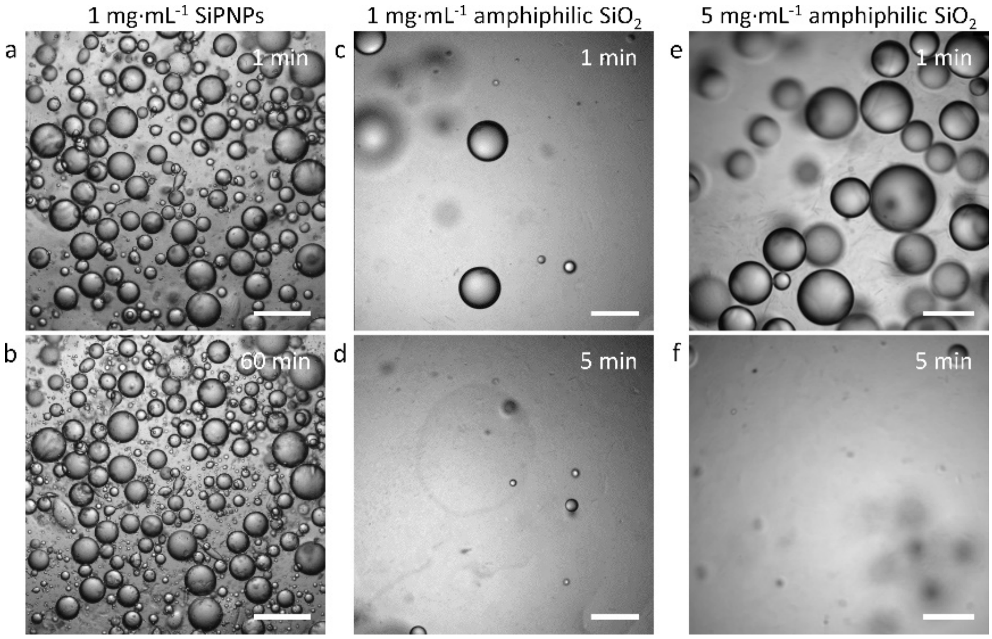

Supplementary Fig. 6. Optical images of isooctanol-in-water emulsions stabilized with 1 mg·mL-1 SiPNPs (a,b), 1 mg·mL-1 amphiphilic SiO_2 (c,d), 5 mg·mL-1 amphiphilic SiO_2 (e,f) with the isooctanol/oil volume ratio of 0.07. The SiPNPs-stabilized emulsion remained stable for more than 60 min (b), while the SiO_2-stabilized emulsions demulsified within 5 min (d, f). Scale bars: 200 μm.

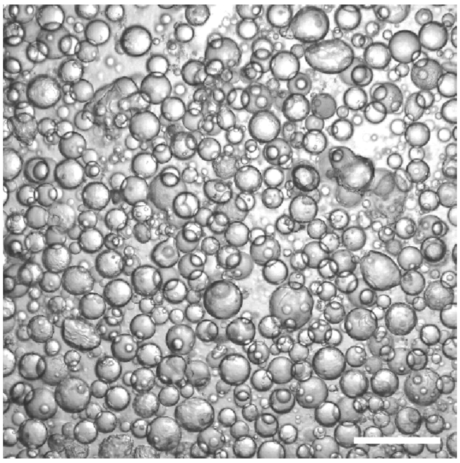

Supplementary Fig. 7. Optical micrograph of crosslinked colloidosomes in isooctanol after 14 days, showing the long-term structural stability. Scale bars: 500 µm.

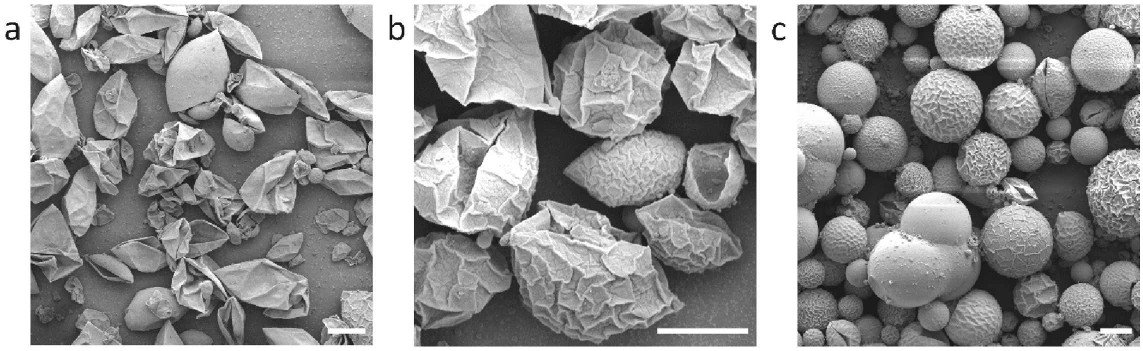

Supplementary Fig. 8. SEM images showing morphological transition of the colloidosomes by adding different amounts of TMOS. TMOS was used as both the crosslinker and the silica precursor. The amount of TMOS per 1.07 mL of colloidosome dispersion was: (**a**) 10 µL, (**b**) 20 µL and (**c**) 30 µL. The images showed that the dried colloidosomes became plumper with more TMOS, probably due to the increased crosslinking between silica particles at higher TMOS concentration, which enhanced mechanical stability. Scale bars: 25 µm.

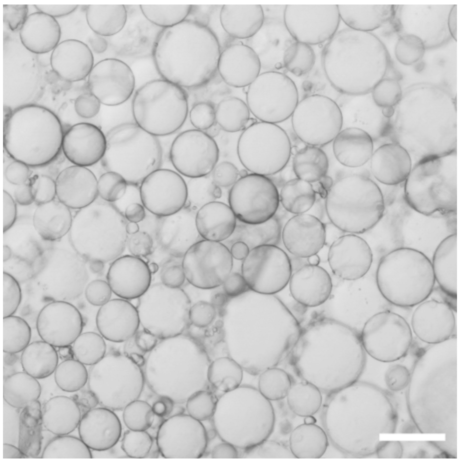

Supplementary Fig. 9. Optical micrograph of crosslinked colloidosomes transferred to the aqueous phase, showing a good structural integrity after phase transfer. Scal bar: 20 μm.

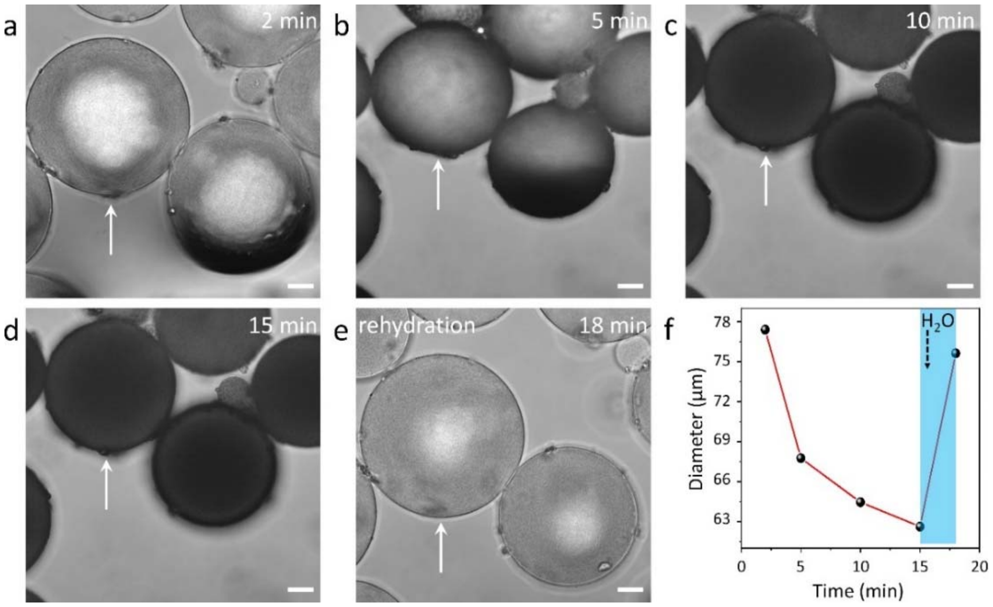

Supplementary Fig. 10. The crosslinked colloidosomes undergoing repeated cycles of dehydration and rehydration. Optical micrographs of the colloidosomes in water after air dry for (**a**) 2 min, (**b**) 5 min, (**c**) 10 min and (**d**) 15 min, showing the microcompartments contracted into approximately 4/5 of the initial size. (**e**) An optical micrograph showing the rehydration of air-dried colloidosomes for 3 min. (**f**) The colloidosomes showed gradual size decrease (~ 20%) after dehydration for 15 min, which were rehydrated to restore their initial size. Scale bars: 20 μm.

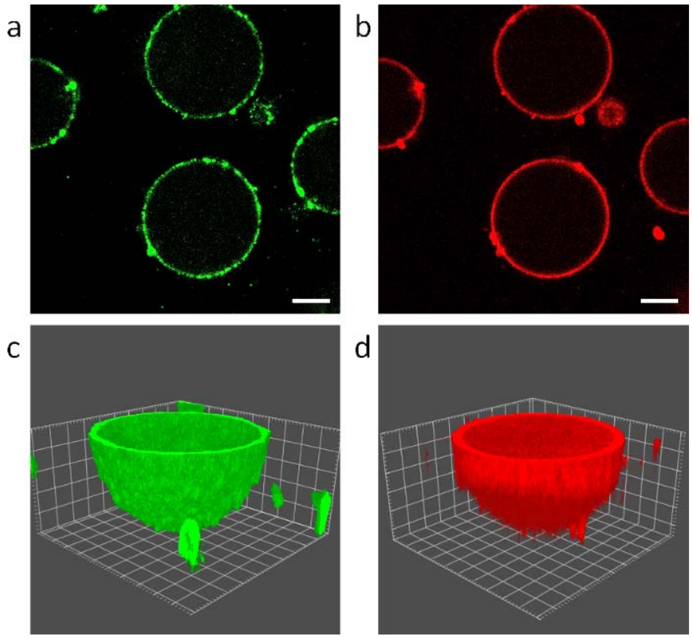

Supplementary Fig. 11. Fluorescence and 3D CLSM images showing the fluorescently labelled layers of the crosslinked three-tiered colloidosomes: (**a,c**) FITC-SiPNPs (10 wt% doped) and (**b,d**) RhITC-lipase (10 wt% doped). The results confirmed the presence of continuous SiPNPs shell (green fluorescence) and lipase inner layer (red fluorescence) at the surface of colloidosomes. Scale bars: 10 μm. Grid widths: 4 μm.

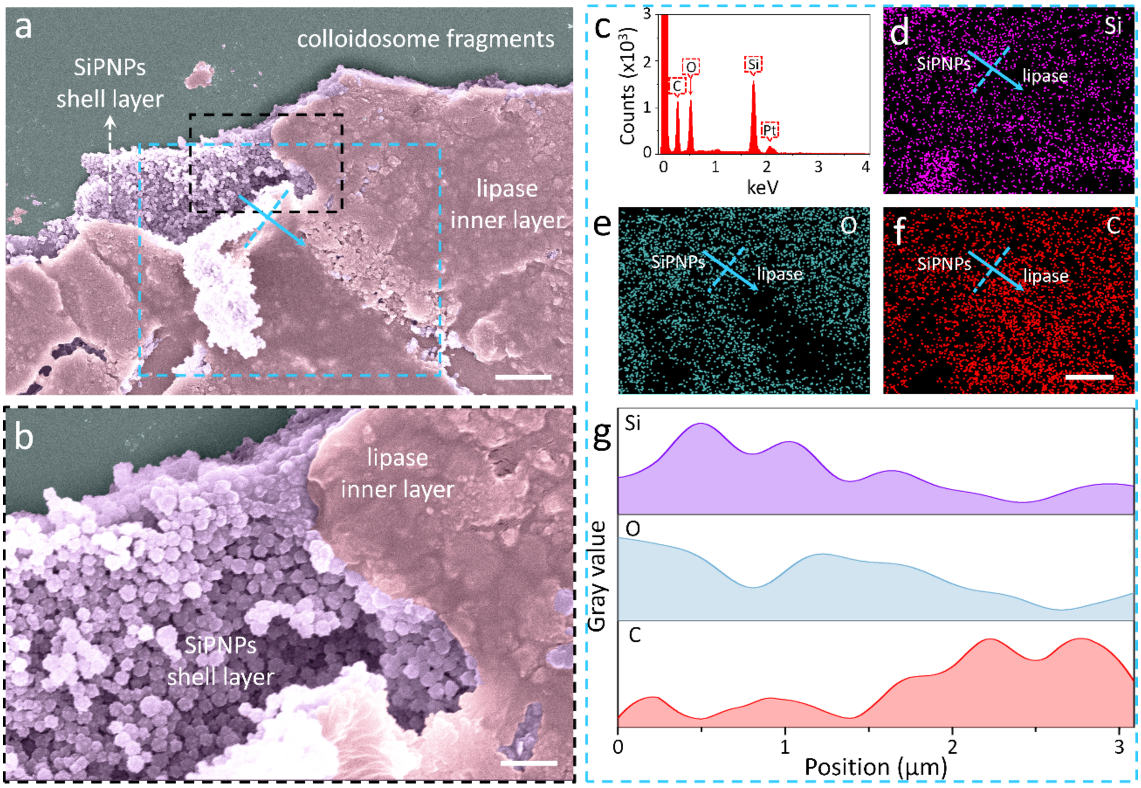

Supplementary Fig. 12. Sectional analysis of a colloidosome consisting of a SiPNPs shell layer and a lipase inner layer. (a) False-colour SEM image of the colloidosome fragments, displaying the SiPNPs outer layer and the peeled lipase inner layer. Scale bar: 2 μm. (b) Magnified false-colour SEM image of the black boxed area of (a), further displaying the adhered SiPNPs outer layer and lipase inner layer. Scale bar: 500 nm. (c-f) Energy dispersive X-ray spectroscopy (EDS) (c) and elemental mapping of Si (d), O (e) and C distribution (f) in the light-blue boxed area of (a). Scale bar: 2 μm. (g) The corresponding EDS line profile analysis indicated by the light-blue lines in (a, d-f), indicating the distinguishable SiPNPs layer and lipase layer with Si and O elemental richness in SiPNPs and that of C element in lipase inner, respectively.

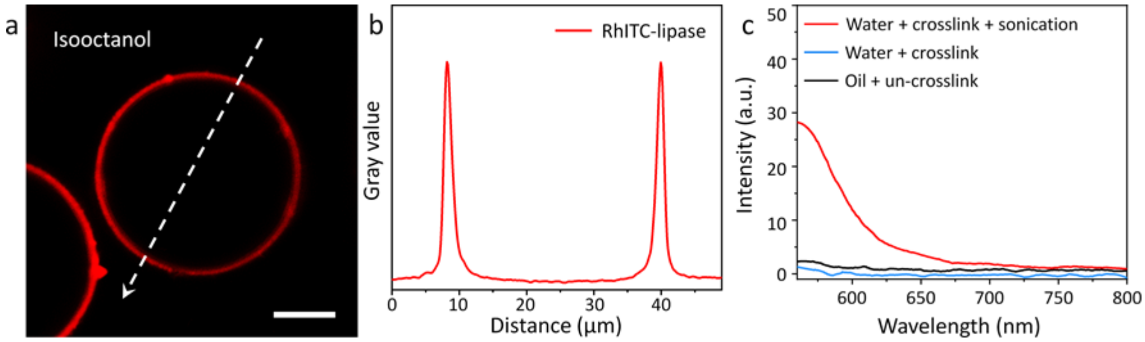

Supplementary Fig. 13. CLSM image (a) and corresponding fluorescence intensity profile (b) of the non-crosslinked colloidosomes in isooctanol, showing the even distribution of RhITC-labelled lipase at the oil-water interface without noticeable leakage. The image was taken 6 h after colloidosome preparation. Scale bar: 20 μm. (c) PL spectra of the supernatant for non-crosslinked colloidosomes in isooctanol, crosslinked colloidosomes in water before and after sonication. The results revealed that lipase was not leaked out unless external sonication was applied, which resulted in the structural destruction of colloidosomes.

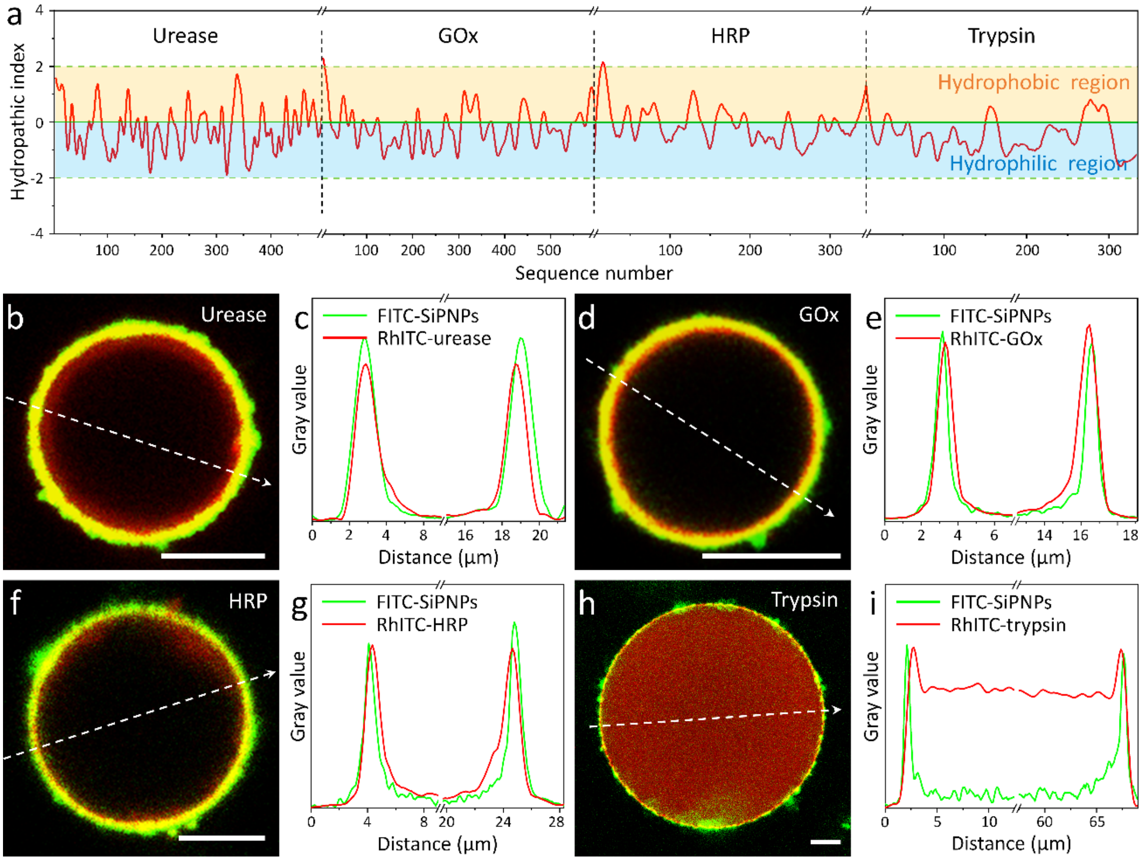

Supplementary Fig. 14. (a) Hydropathic index profiles based on Expasy protein analysis of urease, glucose oxidase (GOx), horseradish peroxidase (HRP) and trypsin, indicating that urease, GOx and HRP contain more hydrophobic residues than trypsin. (b-g) CLSM images and corresponding line profile analysis of three-tiered colloidosomes in isooctanol showing co-assembly of FITC-labelled SiPNPs and amphiphilic enzymes such as rhodamine isothiocyanate (RhITC)-labelled urease (b,c), GOx (d,e), HRP (f,g) at the interface. (h,i) CLSM images and corresponding line profile analysis showing RhITC-trypsin distributed mainly in the lumen, possibly due to the increased hydrophilicity of trypsin. Scale bars: 10 μm.

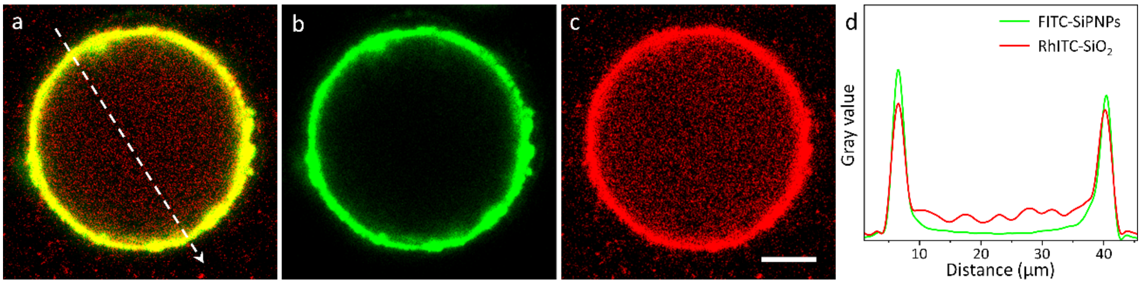

Supplementary Fig. 15. (a-c) CLSM images of the three-tiered colloidosomes doped with the use of FITC-SiPNPs and RhITC-(3-Aminopropyl)triethoxysilane (APTES): (a) overlaid image; (b) green channel; (c) red channel. The yellow ring encapsulating a red lumen (a) suggests the co-localization of SiPNPs (green fluorescence at interface) and newly formed silica (red fluorescence at interface and in lumen), respectively. Scale bar: 10 μm. (d) The corresponding fluorescence intensity profile indicated by the white dashed line in (a) showed the overlaid FITC-SiPNPs and RhITC-SiO_2 on the shell of colloidosomes.

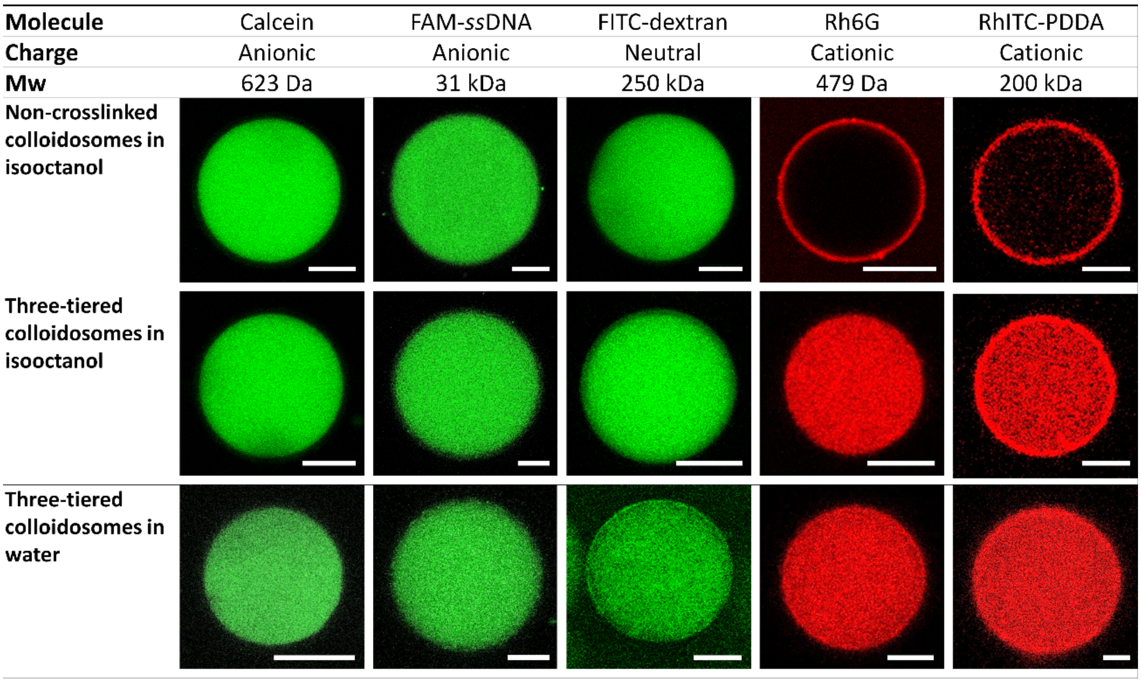

Supplementary Fig. 16. CLSM images for sequestration of molecule inside the non-crosslinked colloidosomes (upper row) and three-tiered colloidosomes in isooctanol (middle row), and three-tiered colloidosomes int water (bottom row). Anionic (calcein, FAM-*ss*DNA) and neutral (FITC-dextran) molecules distributed homogenously in the colloidosomal interior both before and after crosslinking. While cationic molecules (Rh6G, RhITC-PDDA) showed interface preference before crosslinking and redistributed evenly after that. Phase transfer of crosslinked three-tiered colloidosomes from oil phase to aqueous phase for all encapsulation tests did not result in significant changes in molecule fluorescence distributions. Scale bars: 20 μm.

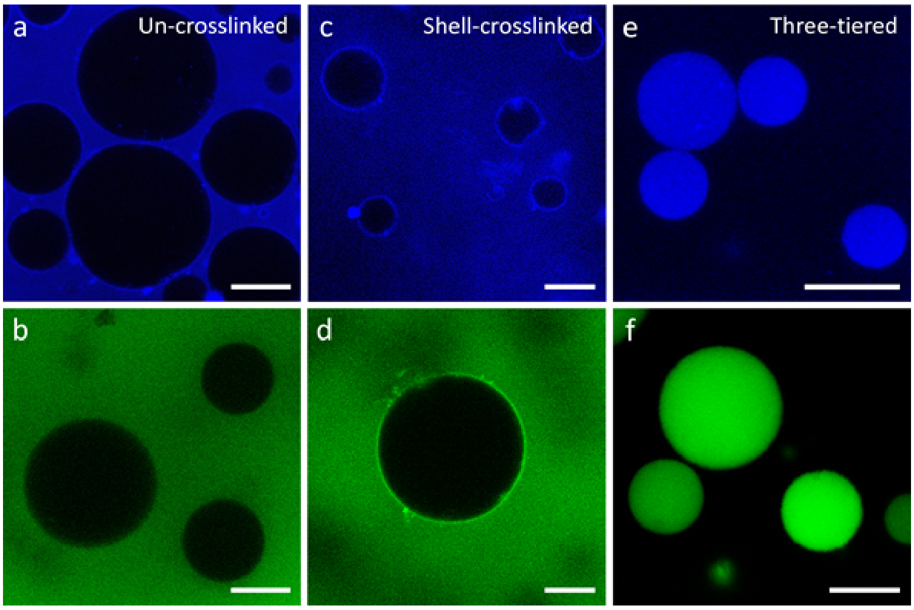

Supplementary Fig. 17. CLSM images showing the uptake of hydrophobic fluorophores by colloidosomes in isooctanol with or without TMOS crosslinking. (**a,b**) Non-crosslinked, (**c,d**) shell-crosslinked (2 µL TMOS) and (**e,f**) three-tiered colloidosomes (20 µL TMOS) after addition of $PyNH_2$ (blue fluorescence) and NBD-PE (green fluorescence), which showed the exclusion of oil-soluble dye molecules for non-crosslinked colloidosomes, while shell-crosslinked colloidosomes can enrich $PyNH_2$ and NBD-PE onto the shell, and the three-tiered colloidosomes exhibited efficient sequestration capability within the lumen. Scale bars: 20 µm

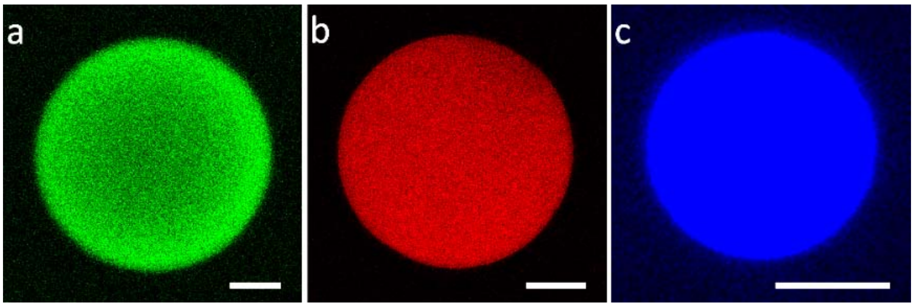

Supplementary Fig. 18. CLSM images showing the uptak of (**a**) calcein (623 Da), (**b**) Rh6G (479 Da), (**c**) Hoechst (534 Da) in the adsorptive aqueous lumen of three-tiered colloidosomes. Scale bars: 10 µm.

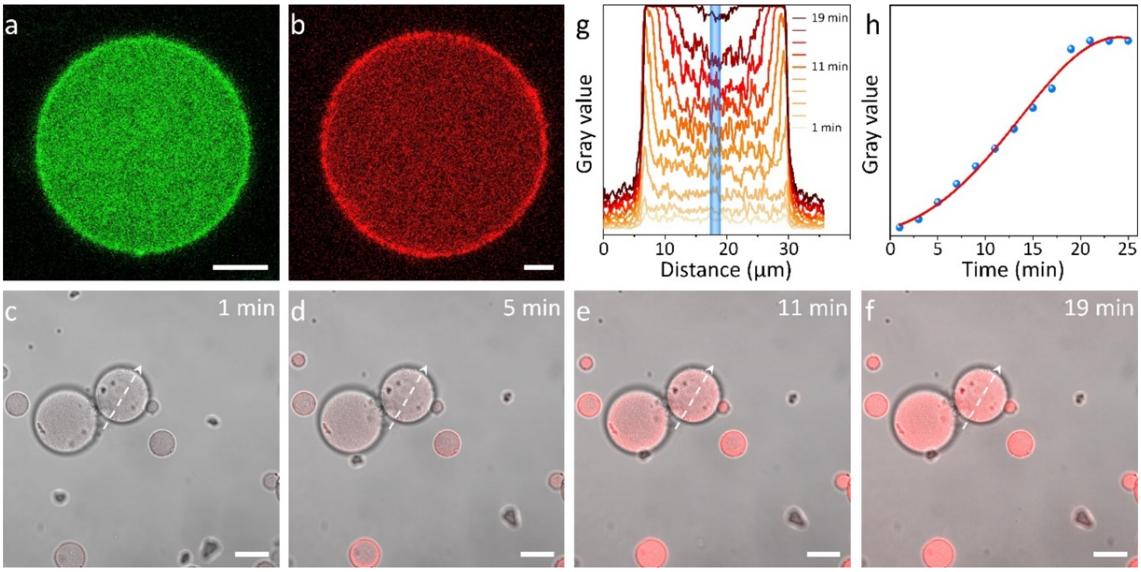

Supplementary Fig. 19. Uptake study of DNA and PDDA by three-tiered colloidosomes in aqueous solution. (**a,b**) CLSM images showing the uptake of (**a**) FAM-ssDNA (31 kDa) and (**b**) RhITC-PDDA (200 kDa). (**c-f**) Overlaid optical and fluorescence microscopy images showing the uptake process of RhITC-PDDA by the three-tiered colloidosomes. Scale bars: 10 μm. (**g**) Time-dependent fluorescence intensity profiles indicated by the dashed lines in (**c-f**), revealing the gradual increase in fluorescence and enrichment of RhITC-PDDA in the colloidosome interior. (**h**) Plot of fluorescence intensity in the center of colloidosome (blue-colored region in **g**) against time, which showed a gradual increase in fluorescence intensity over reaction time.

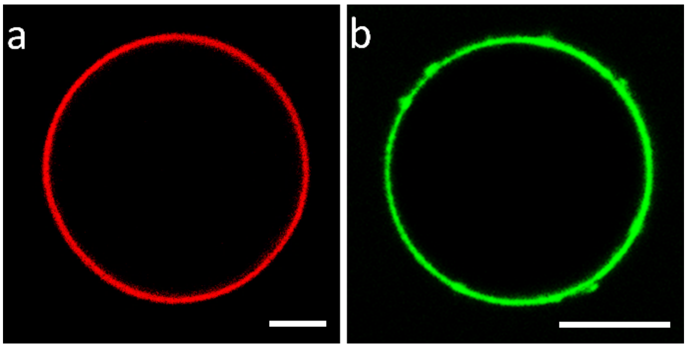

Supplementary Fig. 20. CLSM images showing the localization of fluorescent macromolecules in the presence of three-tiered colloidosomes, (**a**) RhITC-BSA (66.4 kDa) and (**b**) FITC-Dextran (250 kDa). BSA was exclusively located at the surface of colloidosome, while high molecular weight FITC-dextran was excluded by colloidosome. Scale bars: 10 μm.

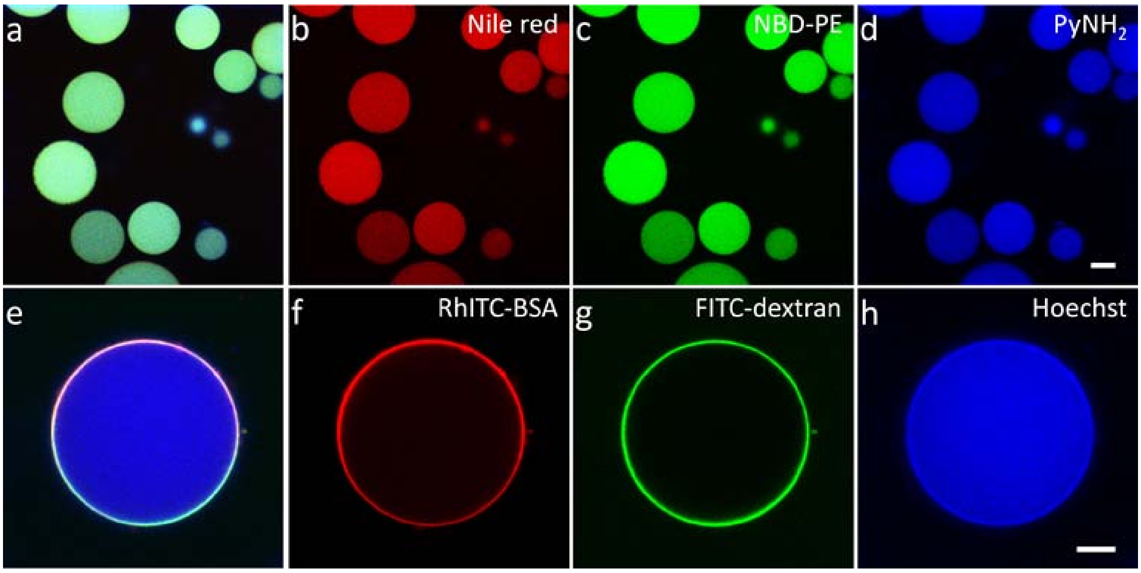

Supplementary Fig. 21. (**a-d**) CLSM image of three-tiered colloidosomes in isooctanol after the addition of mixed hydrophobic dye solution: (**a**) overlaid fluorescence showing a homogenous interior due to the uptake of Nile red (**b**, red), NBD-PE (**c**, green) and $PyNH_2$ (**d**, blue). (**e-h**) CLSM images of three-tiered colloidosomes in water after incubating with mixed water-soluble dye solution: (**e**) overlaid fluorescence showing a discrete red-green ring due to surface adsorption of RhITC-BSA (**f**) and FITC-dextran (**g**), and an encapsulated blue-fluorescent lumen of Hoechst (**h**). Scale bars: 10 μm.

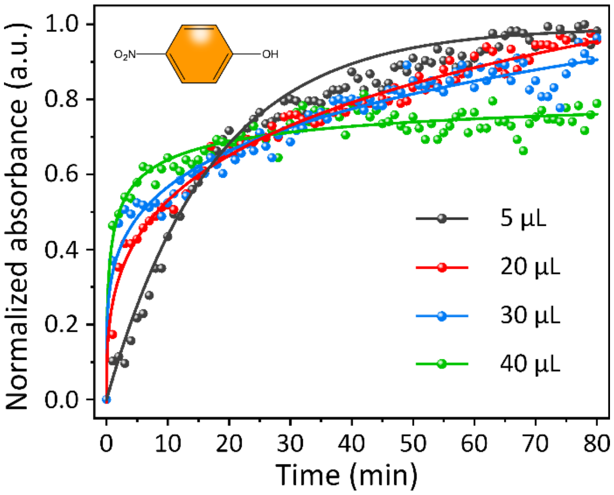

Supplementary Fig. 22. The kinetic plots of p-NPP hydrolysis into p-nitrophenol catalyzed by lipase-loaded colloidosomes crosslinked with 5 µL (black plot), 20 µL (red plot), 30 µL (blue plot) and 40 µL of TMOS (green plot), respectively.

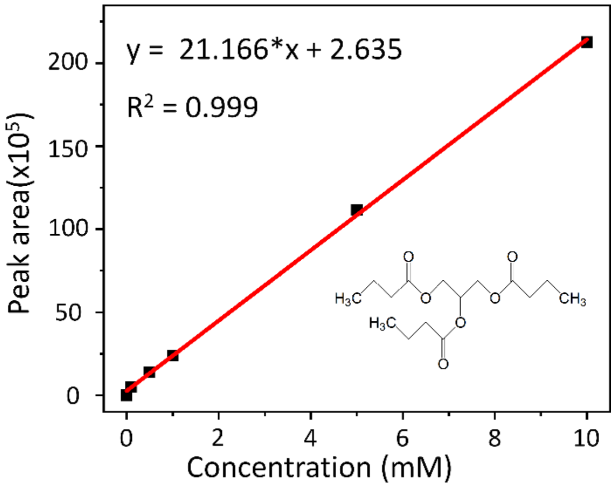

Supplementary Fig. 23. The GTB calibration curve by plotting the GC peak area values (at retention time of 11.93) *versus* concentration in isooctanol. The calibration curve was used to determine GTB concentration in solution during the process of lipase-catalyzed GTB hydrolysis.

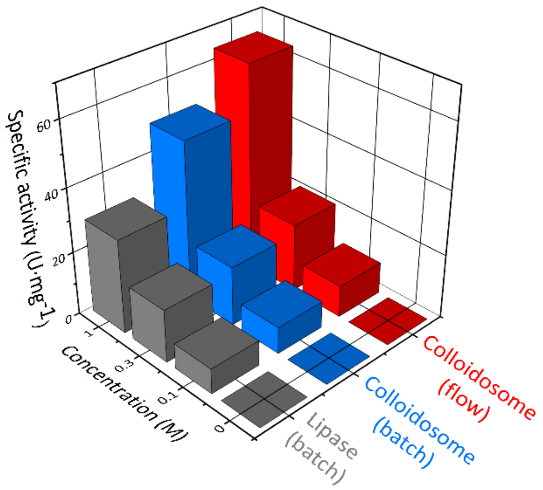

Supplementary Fig. 24. Specific activity of lipase at different concentrations of GTB in the batch reactions within the first 30 min, and in the colloidosome-based flow reaction at steady state (5 mL·h^{-1}), showing the overweighted specific activity of lipase in flow reaction (5 mL·h^{-1}) than batch reactions.

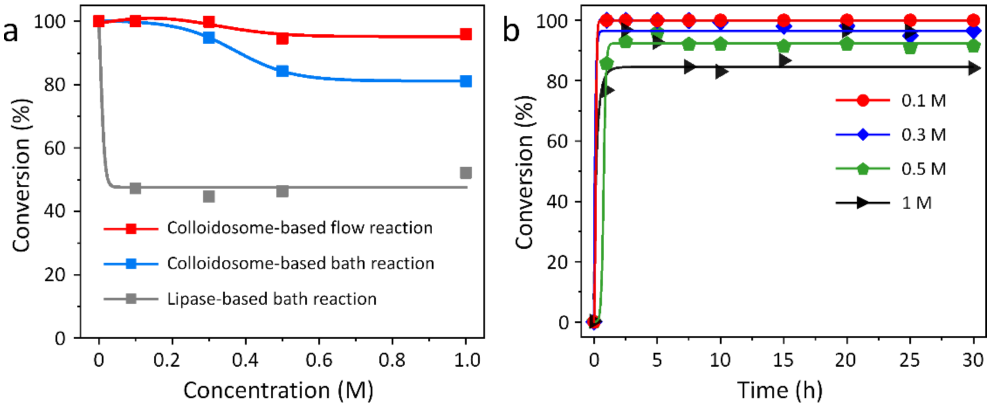

Supplementary Fig. 25. Lipase-catalyzed hydrolysis of GTB with colloidosomes in the continuous flow system. (**a**) GTB conversion versus GTB concentration in the colloidosome-based flow reaction (red plot), colloidosome-based bath reaction (blue plot) and lipase-based bath reaction (gray plot). The data was collected after 20 h reaction, showing higher conversion in continuous flow system over the bath system. (**b**) Time-dependent GTB conversion in the colloidosome-based flow reaction at different GTB concentrations. The continuous flow rate was 1 mL·h^{-1} and the GTB conversion was calculated at steady state.

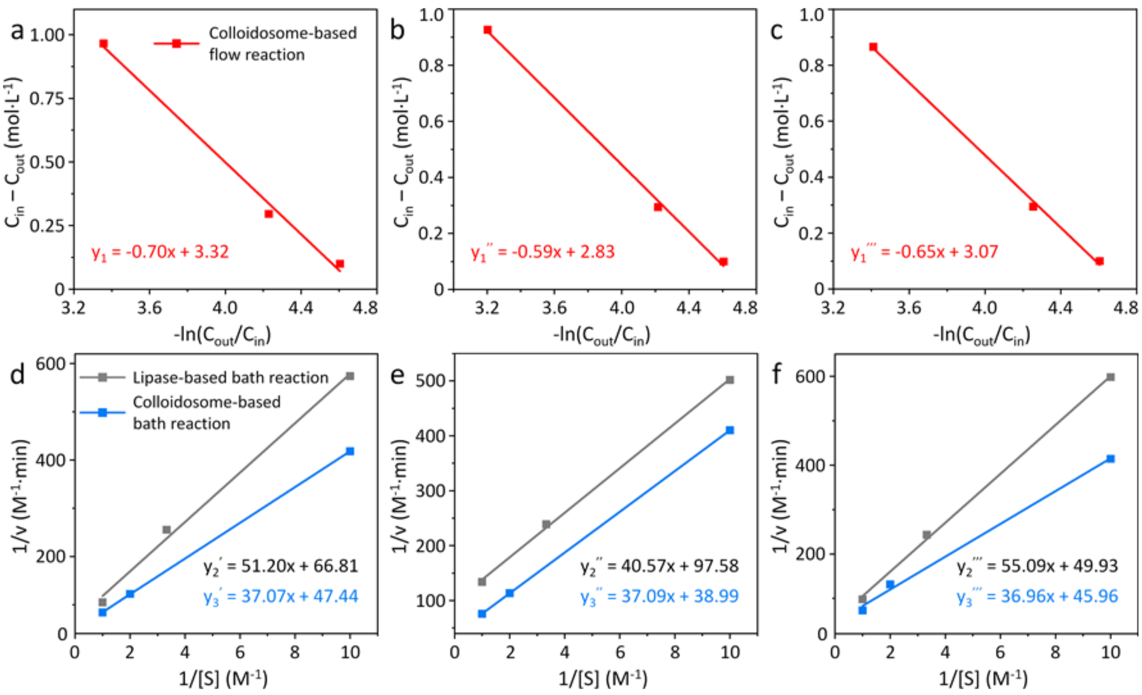

Supplementary Fig. 26. Lilly-Hornby and Lineweaver-Burk plots of lipase-catalyzed GTB hydrolysis. (**a-c**) Lilly-Hornby plot of lipase-catalyzed GTB hydrolysis in colloidosome-based flow reaction (flow rate was 1 mL·h^{-1}). (**d-e**) Lineweaver-Burk plots of GTB hydrolysis in colloidosome-based (blue plot) and lipase-based bath reactions (gray plot). The plots in (**a-c**) and (**d-f**) indicate results derived from three replicating experiments, respectively.

Notes: Kinetic constants of lipase in the continuous flow reaction system were calculated from the Lilly-Hornby model (Lilly M D, Hornby W E, Crook E M. *Biochem. J.* **1966**, 100(3): 718-723; He P, Greenway G, Haswell S J. *Process. Biochem.* **2010**, 45(4): 593-597).

$$C_{in} - C_{out} = K_m \ln\frac{C_{out}}{C_{in}} + \frac{V_{max}V_{void}}{Q}$$

where C_{in} and C_{out} are the substrate concentrations of the influx and efflux, V_{void} is the void volume of the packed column, Q is the flow rate, K_m is the apparent Michaelis constant gained from the slope of the plot, V_{max} is the maximal enzymatic reaction rate calculated from the Y-axis intercept, and the turnover number K_{cat} was obtained according to V_{max}/[lipase].

Kinetic constants of lipase in bath reaction system were calculated from the Lineweaver-Burk equation (He P, Greenway G, Haswell S J. *Process. Biochem.* **2010**, 45(4): 593-597):

$$\frac{1}{v} = \frac{K_m}{V_{max}[S]} + \frac{1}{V_{max}}$$

where ν is the initial enzymatic reaction rate and V_{max} is the maximal one, and $[S]$ is the substrate concentration.

Mass spectra

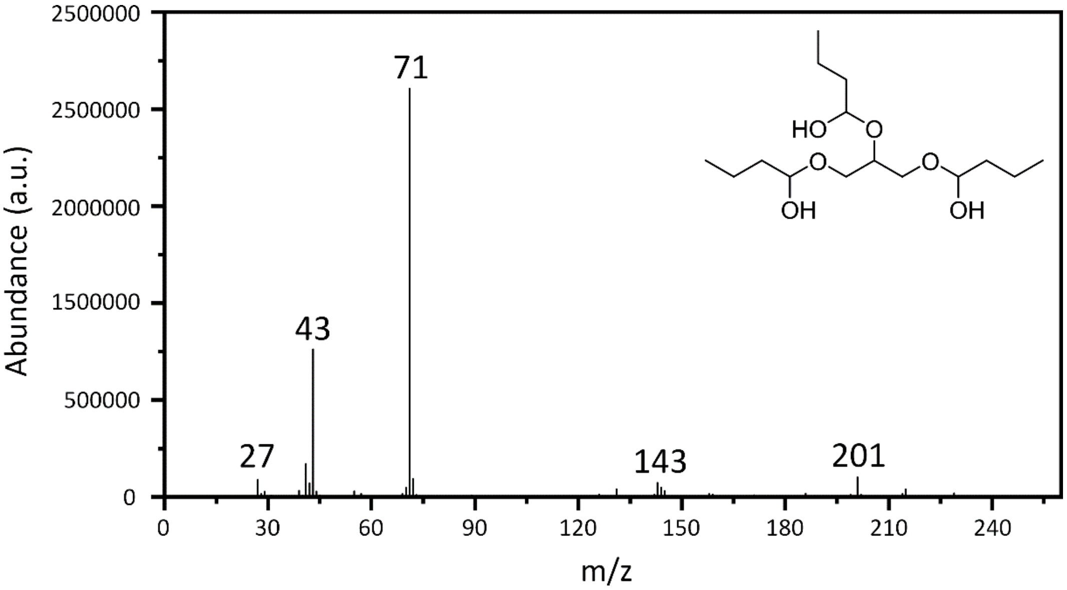

Supplementary Fig. 27. Mass spectra of GTB in isooctanol before the lipase-catalyzed hydrolysis reaction.

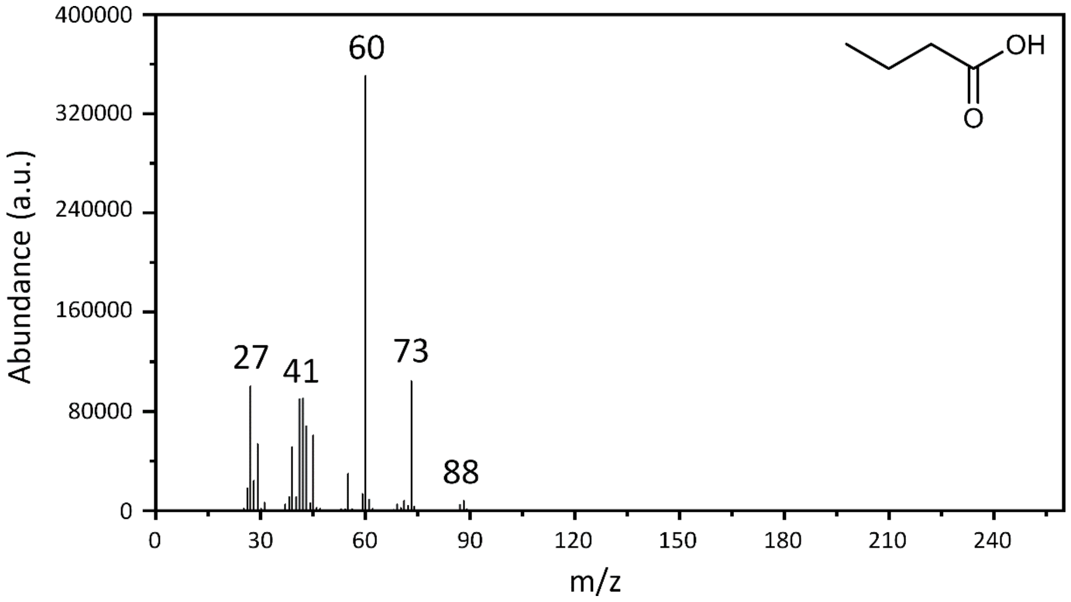

Supplementary Fig. 28. Mass spectra of butyric acid in isooctanol after the lipase-catalyzed hydrolysis reaction.

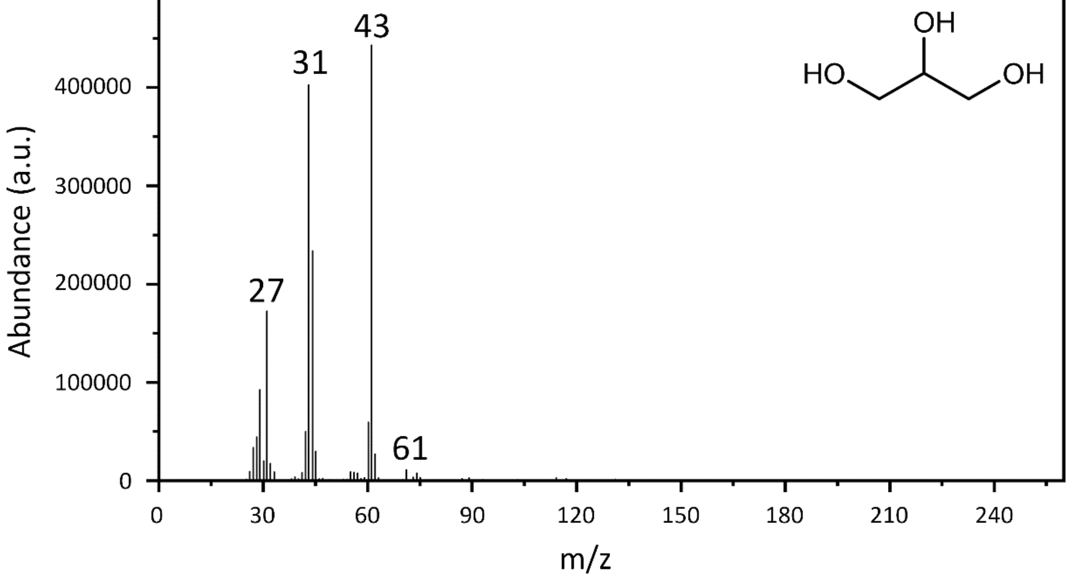

Supplementary Fig. 29. Mass spectra of glycerol in isooctanol after the lipase-catalyzed hydrolysis reaction.

Supplementary Table 1. Oligonucleotide sequences used in *ss*DNA encapsulation and uptake tests of three-tiered colloidosomes.

	Sequence	Modification	Length (Bases)
FAM-*ss*DNA	AAGACTAGAACGAGACTGCCAGGAG TGTAGGACGGAGGTGGAGAATGGATA TGACGGAGGTGCAACGATCACGAAC TTACGAGATGAAACTGCTAATAC	56-FAM at 5’	99
