# Supplementary material for: A three-tiered colloidosomal microreactor for continuous flow catalysis
Source: Nat Commun. 2021 Oct 20;12:6113. doi: 10.1038/s41467-021-26381-x (PMC8528827; doi:10.1038/s41467-021-26381-x)
Supplement: Supplementary file 2 — Description of Additional Supplementary Files [file 41467_2021_26381_MOESM2_ESM.docx]

**Description of Additional Supplementary Files**

**File name: Supplementary Movie 1.**

**Description:** Overlaid fluorescence and optical microscopy video displaying the uptake process of PyNH_2_ by the three-tiered colloidosomes. The blue fluorescence was intensified inside the aqueous colloidosome lumen, revealing the enrichment of oil-soluble PyNH_2_ from the outer isooctanol phase. Movie was shown at ×800 of real-time speed at 20 frames per second.

**File name: Supplementary Movie 2.**

**Description:** Overlaid fluorescence and optical microscopy video revealing the efficient uptake of Hoechst by the three-tiered colloidosomes. The blue fluorescence was intensified inside the aqueous colloidosome lumen, revealing the sequestration of water-soluble Hoechst from the outer aqueous phase. Movie was shown at ×400 of real-time speed at 10 frames per second.

**File name: Supplementary Movie 3.**

**Description:** Overlaid fluorescence and optical microscopy video showing the uptake of RhITC-PDDA by the three-tiered colloidosomes. The red fluorescent water-soluble RhITC-PDDA in external aqueous phase was enriched in the aqueous colloidosome lumen from margin to center, and finally distributed evenly in the whole colloidosome. Movie was shown at ×450 of real-time speed at 20 frames per second.

**File name: Supplementary Movie 4.**

**Description:** Overlaid fluorescence and optical microscopy video displaying the uptake process of RhITC-BSA by the three-tiered colloidosomes. The red fluorescent RhITC-BSA in external aqueous phase was preferentially enriched at the surface of colloidosomes. Movie was shown at ×200 of real-time speed at 10 frames per second.
